# Genomic resolution of an aggressive, widespread, diverse and expanding meningococcal serogroup B, C and W lineage

**DOI:** 10.1016/j.jinf.2015.07.007

**Published:** 2015-11

**Authors:** Jay Lucidarme, Dorothea M.C. Hill, Holly B. Bratcher, Steve J. Gray, Mignon du Plessis, Raymond S.W. Tsang, Julio A. Vazquez, Muhamed-Kheir Taha, Mehmet Ceyhan, Adriana M. Efron, Maria C. Gorla, Jamie Findlow, Keith A. Jolley, Martin C.J. Maiden, Ray Borrow

**Affiliations:** a Vaccine Evaluation Unit, Public Health England, Manchester Medical Microbiology Partnership, Second Floor, Clinical Sciences Building II, Manchester Royal Infirmary, Oxford Road, Manchester, M13 9WZ, UK; b Department of Zoology, University of Oxford, Oxford, UK; c Meningococcal Reference Unit, Public Health England, Manchester Medical Microbiology Partnership, Second Floor, Clinical Sciences Building II, Manchester Royal Infirmary, Oxford Road, Manchester, M13 9WZ, UK; d Centre for Respiratory Diseases and Meningitis, National Institute for Communicable Diseases, National Health Laboratory Service, 1 Modderfontein Road, Sandringham, Johannesburg, 2131, South Africa; e National Microbiology Laboratory, Public Health Agency of Canada, 1015 Arlington Street, Winnipeg, MB R3E 3R2, Canada; f Reference Laboratory for Meningococci, Institute of Health Carlos III, Majadahonda, Spain; g Institut Pasteur, Unité des Infections Bactériennes invasives, Département Infection et Epidémiologie, Paris, France; h Faculty of Medicine, Hacettepe University, Ankara, Turkey; i Servicio Bacteriología Clínica, Departamento de Bacteriología, Instituto Nacional de Enfermedades Infecciosas-ANLIS «Dr. Carlos G. Malbrán», Ciudad Autónoma de Buenos Aires, Argentina; j Division of Medical Biology, Bacteriology Department, Adolfo Lutz Institute, São Paulo 01246-902, Brazil

**Keywords:** Meningococcal, ST-11 clonal complex, Genome, Serogroup W, Serogroup C

## Abstract

**Objectives:**

*Neisseria meningitidis* is a leading cause of meningitis and septicaemia. The hyperinvasive ST-11 clonal complex (cc11) caused serogroup C (MenC) outbreaks in the US military in the 1960s and UK universities in the 1990s, a global Hajj-associated serogroup W (MenW) outbreak in 2000–2001, and subsequent MenW epidemics in sub-Saharan Africa. More recently, endemic MenW disease has expanded in South Africa, South America and the UK, and MenC cases have been reported among European and North American men who have sex with men (MSM). Routine typing schemes poorly resolve cc11 so we established the population structure at genomic resolution.

**Methods:**

Representatives of these episodes and other geo-temporally diverse cc11 meningococci (n = 750) were compared across 1546 core genes and visualised on phylogenetic networks.

**Results:**

MenW isolates were confined to a distal portion of one of two main lineages with MenB and MenC isolates interspersed elsewhere. An expanding South American/UK MenW strain was distinct from the ‘Hajj outbreak’ strain and a closely related endemic South African strain. Recent MenC isolates from MSM in France and the UK were closely related but distinct.

**Conclusions:**

High resolution ‘genomic’ multilocus sequence typing is necessary to resolve and monitor the spread of diverse cc11 lineages globally.

## Highlights

• The meningococcal ST-11 clonal complex is diverse.
• A ‘South American’ serogroup W strain is currently expanding in the UK.
• Supports decision to vaccinate UK teenagers against meningococcal serogroup W.
• A distinct endemic South African strain is related to the 2000 Hajj outbreak strain.
• Serogroup C cases among MSM in UK and France are related but distinct.

## Introduction

The Gram-negative bacterium *Neisseria meningitidis*, the meningococcus, is principally a commensal that colonises the nasopharynx of approximately 10% of humans. It is also a leading cause of meningitis and septicaemia, being associated with sporadic cases, outbreaks and epidemics throughout the world.^1^ The principal meningococcal virulence factor is the polysaccharide capsule which also defines the serogroup. Serogroups A, B, C, W, X and Y are responsible for most disease and vaccines are currently available against serogroups A, C, W and Y.^1^, ^2^ Bexsero, a recently licensed vaccine against serogroup B organisms, targets subcapsular antigens^2^ and was developed in response to safety and efficacy concerns surrounding the B polysaccharide. Reference laboratories conventionally identify serogroups and also type/subtype meningococci on the basis of outer membrane proteins (OMPs). Meningococcal population structure has been studied with multilocus sequence typing (MLST) which classifies meningococci into clonal complexes (ccs). Most invasive isolates belong to a limited number of ccs, which correspond to the hyperinvasive lineages first identified by multilocus enzyme electrophoresis (MLEE).^3^

Meningococci belonging to the ST-11 clonal complex (cc11; also known as the ET-37 complex and lineage 11) are hyperinvasive and may express serogroups C (MenC:cc11) or W (MenW:cc11) and, less frequently, B (MenB:cc11) or Y. They are associated with high rates of morbidity and mortality^4^ and have a propensity to cause outbreaks and epidemics. For example: cc11 meningococci were responsible for serogroup B and C outbreaks in the US military in the 1960s, leading to the implementation of the first generation of meningococcal polysaccharide vaccines.^5^ Epidemics caused by MenC:cc11 in North America, Europe and Australia in the 1990s/2000s, prompted the first use of serogroup C glycoconjugate vaccines^6^ and in 2000 a Hajj-associated outbreak of MenW:cc11 disease swept the globe, persisting for several years.^7^ Since 2001, MenW:cc11 has caused epidemics in the meningitis belt of Sub-Saharan Africa.^8^ North America and Europe have recently experienced several high-profile cases and outbreaks of MenC:cc11 disease among men who have sex with men (MSM), the first being identified in Toronto in 2001.^9^ From 2003, endemic MenW:cc11 disease increased in South Africa, Brazil and, subsequently, several other South American countries where case fatality rates reached 28%.^10^, ^11^ England and Wales have also seen a year-on-year increase in endemic MenW:cc11 disease since 2009.^12^

Beyond the serogroup, currently used typing schemes offer limited resolution among cc11 meningococci. PorB OMP serotypes 2a or NT (non-typable using antisera available from the National Institute for Biological Standards and Control, Potters Bar, UK) are broadly distributed within the cc, whilst almost all MenW:cc11 organisms and a large proportion of MenC:cc11 organisms express the PorA OMP subtype P1.5,2. MLEE was instrumental in identifying the ‘ET-15’ subpopulation responsible for the elevated MenC:cc11 disease of the 1990s/2000s, as well as among MSM cases.^9^ This labour intensive and poorly portable scheme was superseded in late 1998 by MLST^13^; however, the majority of cc11 disease isolates exhibit a single sequence type (ST-11). PorA also poorly resolves these meningococci as, although subtypes P1.5-1,10-8 and P1.5-1,10-4 are the most common, a large proportion exhibit the widespread P1.5,2 subtype.^14^ Consequently, research groups and reference laboratories have targeted individual ET-15 markers such as the genomic presence of the insertion sequence IS*1301* or the fumerase (*fumC*) gene mutation responsible for the characteristic electropherotype.^15^, ^16^ As none of the typing markers employed to date are satisfactory, there is uncertainty as to the relatedness of current and historical cases and the provenance of emerging cc11 strains. Isolates typed as MenW:cc11, for example, are often described as ‘the Hajj strain’ with no direct evidence that they are closely related.^17^

Whole genome sequencing (WGS) of meningococcal genomes is currently the most cost effective method for meningococcal typing for surveillance, especially with the advent of protein-based serogroup B vaccines that include antigens not historically used for typing.^2^ The Meningitis Research Foundation Meningococcus Genome Library (MGL) is an exemplar of this concept, containing genomes for all English and Welsh and Northern Irish invasive isolates received by the Public Health England Meningococcal Reference Unit (MRU) from July 2010 to June 2013 (inclusive). These high-resolution data facilitate surveillance and the identification of outbreaks. The power of the MGL is enhanced by its capacity to compare current data to historical and international cases within the broader PubMLST *Neisseria* database – an open access repository of Neisserial isolate data including provenance, pheno/genotyping data and, increasingly, whole genome sequences, submitted by researchers and reference laboratories around the world (http://pubmlst.org/neisseria/). The elevated levels of disease caused by cc11 and the historical significance of these hyperinvasive meningococci make them a priority for such an analysis. Here we investigated an extensive panel of cc11 isolates, including representatives of high-profile disease episodes from the present and recent past, to address these issues.

## Methods

### Isolates and genomes

All genomes are available open access in the PubMLST *Neisseria* database (accessed 29 October 2014). Unique PubMLST IDs and typing data are listed in Supplementary Table 1. Genomes of 448 of the isolates were sequenced, assembled and annotated as part of the present study, as described previously.^18^ The isolates covered in the study comprised a mixture of MGL isolates, isolates from noteworthy episodes of cc11 disease (mainly sequenced for the purpose of the study) and other available cc11 genomes on the PubMLST *Neisseria* database (Supplementary methods).

Briefly, the study included genomes for 750 cc11 isolates comprising 246 serogroup C isolates, 470 serogroup W isolates and 34 serogroup B isolates. These included 663 invasive disease isolates, 40 upper respiratory tract carriage isolates and 47 isolates from unspecified or unknown sites. There were 522 isolates from the UK and 228 representing 24 other countries across five continents and several decades (Supplementary Table 2) including MenW:cc11 isolates from English, Welsh and French Hajj pilgrims (2000–2004), North Africa (1996–2004), South Africa (2003–2013), Brazil (2008–2011), Argentina (2008–2012) and England and Wales (2009–2014); ET-15 and non-ET-15 MenB:cc11 and MenC:cc11 isolates from Europe and North America from the mid 1990s to the present; and MenC:cc11 isolates from MSM cases in Toronto (2001),^19^ England (2011–2013) and France (2013).^20^

Two diverse non-cc11 isolates (PubMLST IDs 29645 (cc8) and 27778 (cc41/44)) were included in order to identify a putative origin for the cc11 lineage.

### Phylogenetic analyses

Genomes were compared using the PubMLST Genome Comparator tool,^21^ which employs a ‘gene by gene’ approach comparing arbitrary, sequentially assigned, pre-indexed allele identifiers at each locus.^18^ A set of 1546 indexed core genome (cg) loci were used for comparisons as described previously.^12^ SplitsTree4 (version 4.12.8) was used to visualise the resulting distance matrices as Neighbour-net networks.^18^, ^22^

### Role of the funding sources

The funding sources for the study had no role in study design, data collection, data analysis, data interpretation, or writing of the report. The corresponding author had full access to all the data in the study and had final responsibility for the decision to submit for publication.

## Results

**Figure 1:**
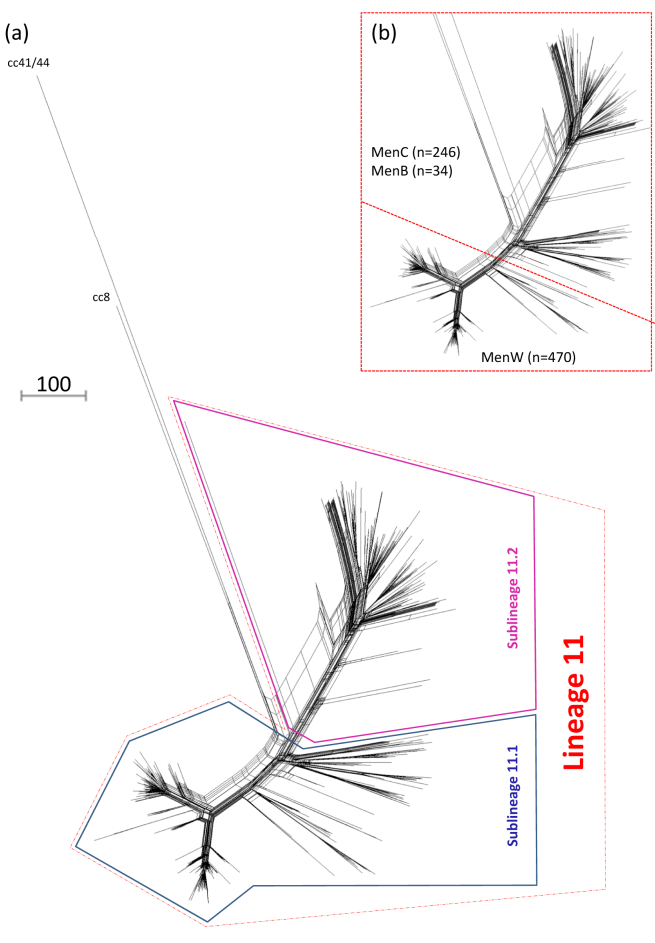
Population structure and serogroup distribution among geographically and temporally diverse meningococcal cc11 isolates versus non-cc11 isolates. (a) Neighbour-net phylogenetic network based on a comparison of 1546 core genome loci among geographically and temporally diverse cc11 isolates (n = 750) and two non-cc11 isolates (cc8 and cc41/44, respectively). The cc11 isolates bifurcated into two main sublineages (lineages 11.1 and 11.2), each containing several clusters of isolates. Panel (b) illustrates the serogroup distribution whereby serogroups B (MenB) and C (MenC) were present in both sublineages while serogroup W (MenW) isolates were confined to the intermediate and distal regions of lineage 11.1. The scale bar indicates the number of differences among the 1546 loci compared.

A comparison of all of the genomes demonstrated a relatively close relationship between the cc11 and cc8 genomes versus that of the cc41/44 isolate (Fig. 1a). The cc11 genomes formed a single main lineage comprising two sublineages, lineage 11.1 and lineage 11.2, bifurcating from the non-cc11 genomes. Both sublineages gave rise to several clusters, each representing periods of putative clonal expansion. The serogroup W isolates were confined to several relatively distal clusters within lineage 11.1 whilst the serogroup B and C isolates were confined to the remaining lineage 11.1 clusters and all of lineage 11.2 (Fig. 1b).

### Lineage 11.2 – the ‘ET-15 lineage’

**Figure 2:**
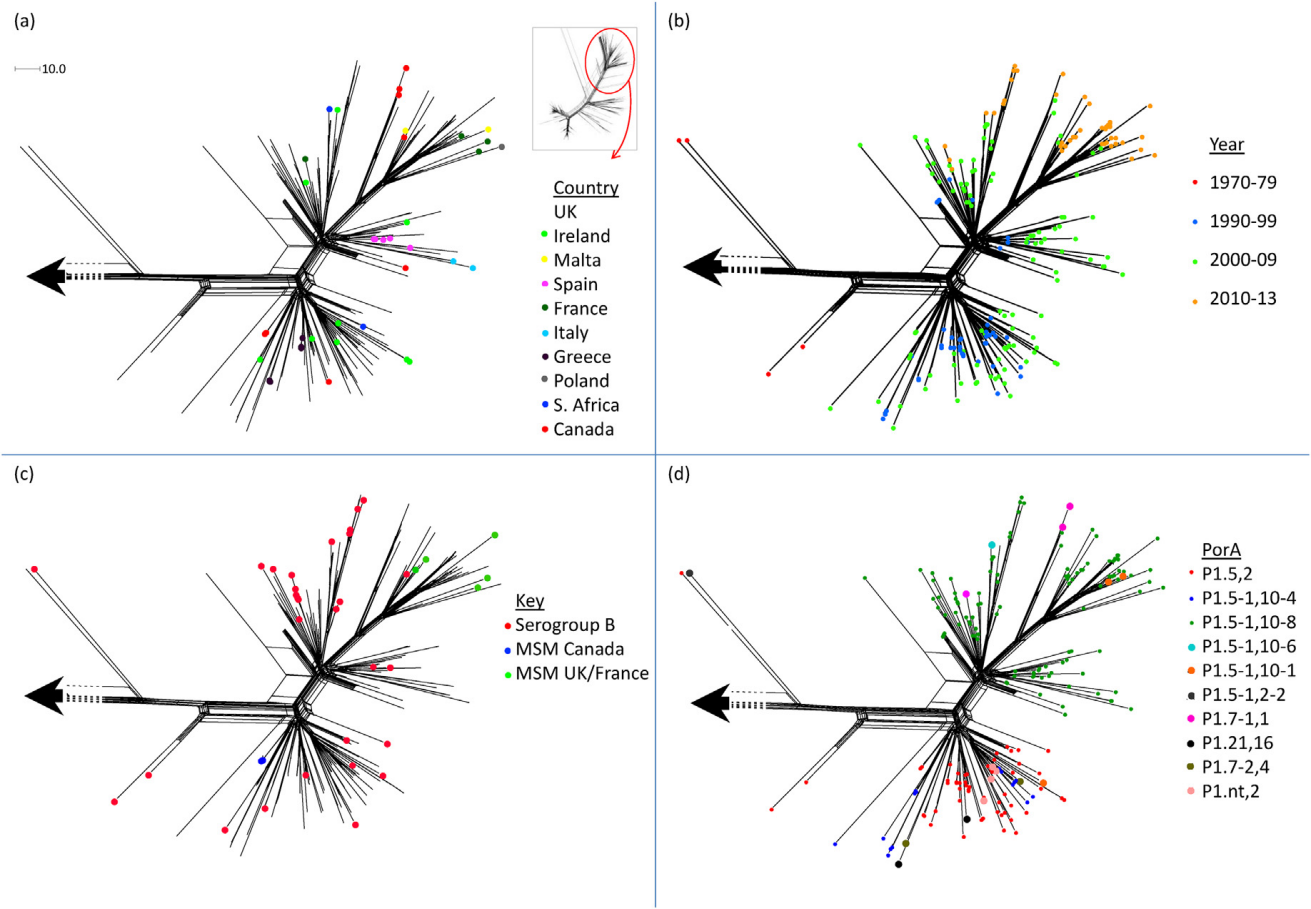
Geo-temporal, serogroup and serotype distributions of meningococcal lineage 11.2 isolates and the distribution of invasive isolates from British and French men who have sex with men. The inset in section (a) indicates the region of cc11 represented by lineage 11.2. The corresponding isolates were compared, along with 71 diverse representatives of lineage 11.1 (Supplementary Table 1 – highlighted yellow), in terms of 1546 core genome loci to generate the Neighbour-net phylogenetic network in sections (a) to (d). The large arrow represents the lineage 11.1 isolates. (a) The relatively populous UK isolates (no marker) were broadly interspersed among isolates from countries representing several continents. (b) The diversification of the sublineage was reflected in the temporal distribution of the isolates, with more recent isolates often observed at the more distal regions of the lineage. Section (c) highlights the distribution of (i) serogroup B isolates which were highly interspersed among the serogroup C isolates (no markers), (ii) MenC:cc11 genomes from the Toronto MSM outbreak (Canada MSM), and (iii) the relatively diverse recent UK and French MSM cases that were interspersed with cases from the wider community. Section (d) depicts the distribution of PorA subtypes in which the isolates in the relatively proximal clusters (versus the origin of the sublineage) were mainly P1.5,2 or P1.5-1,10-4, whilst isolates within more distal clusters were mainly P1.5-1,10-8. The scale bar in section (a) indicates the number of differences among the 1546 loci compared.

Lineage 11.2 comprised solely serogroup B and C isolates. Isolates from several European countries, South Africa and Canada were well distributed within the sublineage (Fig. 2a). To our knowledge, all of the known ET-15 isolates (as determined by MLEE) were found in lineage 11.2, as were all isolates possessing the characteristic ET-15 *fumC* polymorphism and the majority of isolates with the cc11/ET-15 marker, IS*1301*. Only three isolates (those nearest to the origin of the sublineage) lacked both of the latter ET-15 markers. A further isolate within the mainly PorA P1.5,2 cluster (PubMLST ID 1178) lacked the *fumC* polymorphism. The temporal distribution of the isolates largely reflected the longitudinal expansion of the population over time with the earliest isolates relatively close to the origin of the sublineage whilst the distal portion was dominated by the most recent isolates (Fig. 2b). The serogroup B and C isolates were broadly interspersed throughout the sublineage (Fig. 2c). The Canadian MSM outbreak isolates were almost identical to one-another whilst the recent English and French MSM isolates were relatively distinct from one another and were interspersed among sporadic cases in the wider community (Fig. 2c). Notably, however, the French and English MSM isolates shared a relatively recent common ancestor within the most distal cluster. Isolates with PorA P1.5,2 dominated the clusters closest to the origin of the sublineage giving way to P1.5-1,10-8 in the more distal clusters (Fig. 2d).

### Lineage 11.1

#### Lineage 11.1 (non-ET-15) serogroup B and C isolates

The remaining serogroup B and C isolates, including most of the isolates from pre-1990 (17/21), were confined to several closely related clusters and singletons located near to the origin of lineage 11.1 (Fig. 1a and b, Supplementary Table 1 ‘lineage 11.1 proximal region’). In the current collection, most of these serogroup C isolates were from the UK (n = 49/65; 1970–2014), but they also included isolates from Ireland (n = 5, 2001–2011), Malta (n = 2, 2006–2013), Spain (n = 1, 1985), Italy (n = 1, 1984), Israel (n = 1, 1988), Ghana (n = 1, 1984), Mali (n = 1, 1989), Brazil (n = 1, 1976), Canada (n = 2, 2008–2009), and the USA (n = 1, 1983). The majority of these (47/65) possessed PorA P1.5,2, with P1.5,2-1 (n = 12) predominating in a single cluster that included isolates from the UK (n = 8, 1985–2005), Malta (n = 1, 2006), Spain (n = 1, 1985), Israel (n = 1, 1988) and Ghana (n = 1, 1984). Most of the isolates from 2007 onwards (19/21) were found in a single cluster comprising isolates from the UK (n = 18, 1998–2014), Ireland (n = 1, 2011), Malta (n = 1, 2013), and Canada (n = 2, 2008–2009). Four serogroup B isolates in this region were interspersed among the serogroup C isolates and were from England and Wales (2001 and 2012), Norway (1969), and the USA (1964).

#### Lineage 11.1 serogroup W isolates

The serogroup W isolates were exclusively located in the relatively intermediate and distal regions of lineage 11.1, where they were segregated from the serogroup B and C isolates (Fig. 1a and b). In the intermediate region were two clusters of South African isolates (n = 15, 2003–2013; and n = 9, 2003–2011), a cluster of UK isolates (n = 6, 1975–1990), and several unclustered UK isolates (n = 8, 1996–2007). The latter South African cluster comprised all PorA P1.5-1,2 isolates whilst the majority of all of the remaining serogroup W isolates (452/471) were P1.5,2.

**Figure 3:**
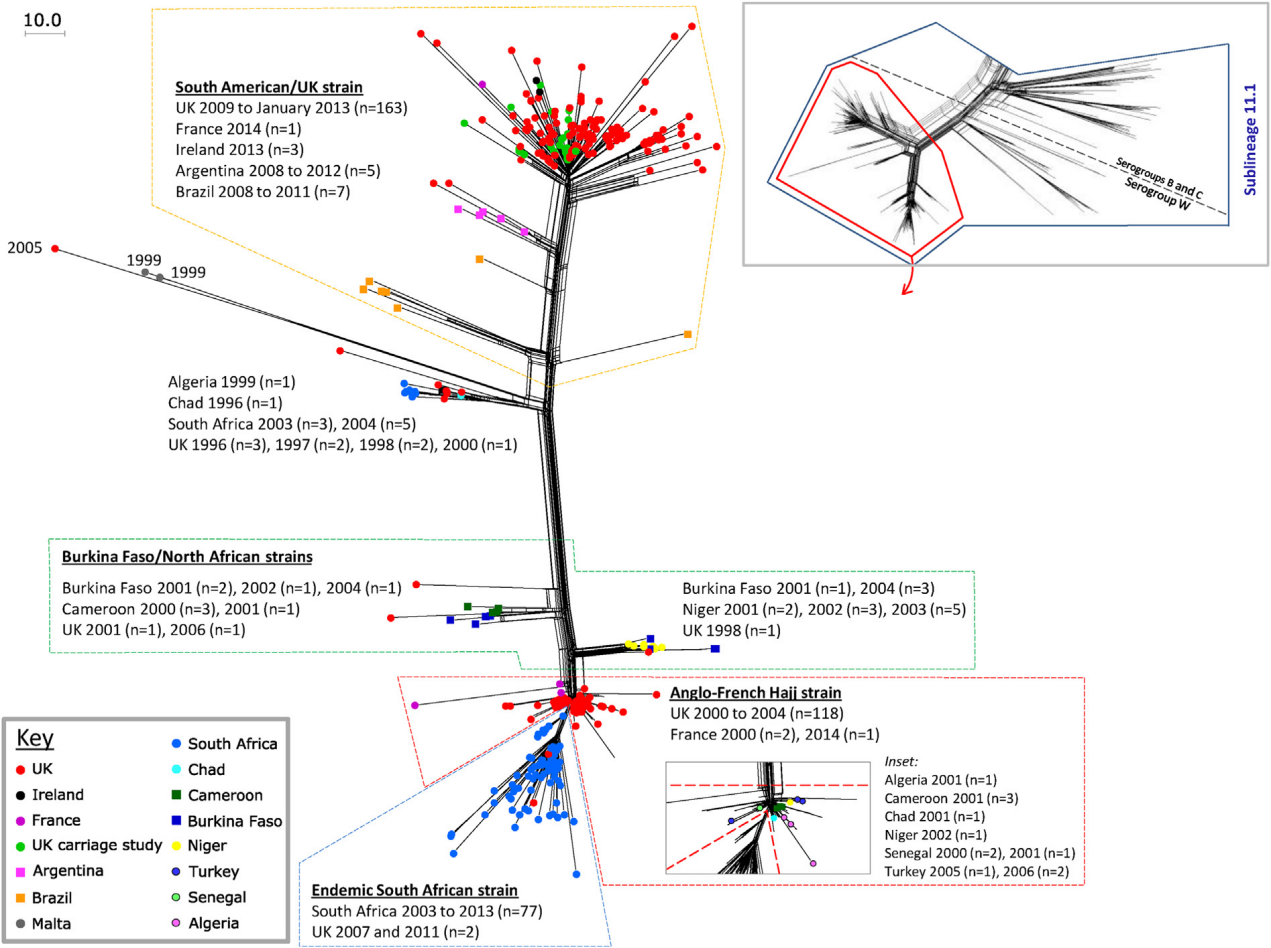
Geo-temporal distribution of isolates within distal sublineages of meningococcal lineage 11.1. The inset (top-right) depicts a cgMLST (1546 loci) Neighbour-net phylogenetic network of all 750 geo-temporally diverse cc11 isolates and two non-cc11 isolates (cc8 and cc41/44) highlighting the distal region of lineage 11.1 that bifurcates into two sublineages. Isolates corresponding to this region underwent a separate cgMLST (1546 loci) comparison to generate the Neighbour-net network in the main figure. Both sublineages contained several clusters, each relating to a noteworthy episode of serogroup W disease. One lineage included strains relating to the Hajj outbreak of 2000 onwards (Anglo-French Hajj strain), the expansion of endemic MenW:cc11 disease in South Africa from 2003 (endemic South African Strain) and a period of MenW:cc11 epidemics in sub-Saharan Africa (Burkina Faso/North African Strains). The other sublineage contained clusters relating to expanding endemic MenW:cc11 disease in South America and the UK (the South American/UK strain). Dots relate to individual cases. The scale bar indicates the number of loci differing among the 1546 compared.

The distal region of lineage 11.1 bifurcated into two main sublineages and a small intermediate cluster, the latter comprising isolates from the UK (n = 8, 1996–2000), South Africa (n = 8, 20032004), Algeria (n = 1, 1999) and Chad (n = 1, 1996) (Fig. 3). Within one of the two main sublineages was a tight cluster including the two French Hajj pilgrim isolates and almost all (118/120) of the English and Welsh MenW:cc11 isolates from the Hajj outbreak period (2000–2004), including those of 14 pilgrims and 41 pilgrim contacts. These were accompanied by several North African isolates from the same period, and more recent isolates from Turkey (n = 3, 2005/6) and France (n = 1, 2014) (Fig. 3, ‘Anglo-French Hajj strain’). Slightly more distal within the same sublineage was the main South African isolate cluster comprising 79/112 South African isolates (2003–2013) and two English isolates (2007 and 2012) (Fig. 3, ‘endemic South African strain’). Towards the origin of this sublineage were two further clusters, respectively comprising isolates from Burkina Faso (n = 3, 2001–2004), Niger (n = 10, 2001–2013) and the UK (n = 1, 1998), and Burkina Faso (n = 4, 2001–2004), Cameroon (n = 4, 2000–2001) and the UK (n = 1, 2001) (Fig. 3, ‘Burkina Faso/North African isolates’).

The other main sublineage in the distal region of lineage 11.1 contained a large cluster comprising invasive isolates from England and Wales (n = 117, 2009–2013), Ireland (n = 3, 2013) and France (n = 1, 2014), as well as 29 MenW:cc11 carriage isolates from five of the six universities partaking in a recent UK carriage study.^23^ Towards the origin of this sublineage were two further clusters, respectively comprising isolates from Argentina (n = 5, 2008–2012) and England and Wales (n = 2, 2010–2014); and Brazil (n = 6, 2008–2011). A further isolate from Brazil was located between the latter two clusters (Fig. 3, South American/UK strain).

Ten serogroup W isolates possessed IS*1301* – four ‘South American/UK strain’, five ‘endemic South African strain’ (of which one was isolated in England/Wales), and one ‘Anglo-French Hajj strain’ (isolated in Turkey 2006).

## Discussion

For at least fifty years, isolates belonging to meningococcal cc11 have been responsible for outbreaks and epidemics of invasive meningococcal disease. The most recent concern is a possible international outbreak of MenC:cc11 disease among MSM and the demonstrated expansion of MenW:cc11 disease in England and Wales, South Africa and South America where associated case fatality rates have been particularly high. The poor discriminatory value of routine typing schemes against cc11 isolates has led to uncertainty as to the relatedness of current and historical cases and the provenance of emerging strains. In the present study, WGS data from several countries over several decades were compared to generate a high resolution framework for cc11 epidemiology.

The present analysis confirmed the previously described close relationship between cc11 and cc8 meningococci.^24^ The cc11 isolates comprised two major sublineages (lineage 11.1 and lineage 11.2) diverging from the non-cc11 isolates, as has previously been shown with fewer isolates.^18^, ^25^ The serogroup B and C isolates were present in both sublineages with the confirmed ET-15 isolates and all isolates possessing the characteristic ET-15 *fumC* polymorphism confined to lineage 11.2. An isolate located deep within lineage 11.2 lacked the *fumC* mutation, probably through recombination which is common in meningococci. The majority of isolates possessing IS*1301* were also found in this sublineage, though a diverse set of lineage 11.1 isolates also appear to have acquired the insertion sequence following the divergence of the two sublineages. Three further lineage 11.2 isolates lacking these ET-15 markers were located close to the origin of lineage 11.2 indicating that the markers became ‘fixed’ sometime after the initial divergence of lineages 11.1 and 11.2.

The results were consistent with serogroup B and C capsular switching events^26^ occurring on multiple occasions, rather than a small number of rare events giving rise to several persistent serogroup B strains. The relatively large distances observed among the English/Welsh and French MSM isolates, and the fact that they were interspersed among sporadic community cases, suggest that these did not constitute an outbreak comprising a close transmission network, as was observed among the epidemiologically linked Toronto cases for which the isolates were practically identical to one-another.^19^ The distribution of P1.5-1,10-8 among our panel suggests that other recent MSM isolates with this PorA subtype would also fall into these closely related clusters. Inclusion of such isolates in future analyses will establish the extent and distribution of outbreaks. It remains possible that isolates located in more distal clusters of lineage 11.2 have a genuine association with disease in this community, perhaps due to a propensity to colonise a genitourinary niche, although this remains to be established.^9^

All of the serogroup W isolates were located in lineage 11.1 and were distinct from the serogroup B and C isolates. The lineage 11.1 cluster that included invasive isolates from Hajj pilgrims (the ‘Anglo-French Hajj strain’) can be considered a Hajj-associated outbreak strain. Analysis of genomes of Hajj outbreak isolates from other countries including Saudi Arabia will confirm whether this was responsible for the wider outbreak or there was a concurrent outbreak due to a distinct strain. It is possible that the North African isolates belonging to this cluster also formed part of the Hajj outbreak, thereby supporting a ‘single origin’ outbreak. Conversely, they may represent an endemic African strain from which the outbreak arose: all of these isolates were listed as sporadic cases except one that was listed as epidemic. The predominance of North African isolates in two closely related, but relatively less distal, clusters adds support to this contention. These included four isolates each from Burkina Faso between 2001 and 2004 (each listed as ‘epidemic’). Thus these and the Anglo-French Hajj strain appear to have diverged from a single, relatively recent ancestor. This also highlights the presence of at least two MenW:cc11 strains in Burkina Faso at the time of the large MenW:cc11 epidemics,^8^ neither of which corresponded to a confirmed Hajj strain. The related UK strain from 2001 was confirmed to have no epidemiological link to the Hajj outbreak.

The South African MenW:cc11 isolates were diverse, being located in several clusters in the distal and intermediate regions of lineage 11.1. The predominant cluster, however, was very closely related to, and apparently divergent from, the Anglo-French Hajj strain. Given that the increase in endemic MenW:cc11 disease in South Africa began in 2003^10^ (shortly after the onset of the Hajj outbreak in 2000), it is likely that this strain was introduced during or shortly after the outbreak, at which point it became endemic for several years. The Anglo-French Hajj strain was also detected in more recent isolates from France (2014) and Turkey (2005/6) where it has been speculated to persist as an endemic strain.^27^

Importantly, this WGS analysis demonstrates that the MenW:cc11 strain which is currently endemic in Brazil, Argentina and England and Wales, the ‘South American/UK strain’, is distinct from the Hajj outbreak strain. It is also epidemiologically distinct with a tendency to persist, as compared with the Anglo-French Hajj and South African strains for which elevated disease rates subsided relatively quickly.^7^ Despite the comprehensiveness of the panel of recent English and Welsh MenW:cc11 case isolates, the South American/UK strain was not observed in these two countries prior to 2008. It is interesting that the positions of the Brazilian, Argentinean and English/Welsh clusters, relative to the origin of the sublineage, reflected the timeline in which each country began to experience increased disease. Also interesting is the fact that isolates from two English cases (2010 and 2014) formed a cluster with the Argentinean isolates. These observations are consistent with the suggestion that this strain has spread recently from South America and become endemic in England and Wales. Furthermore, one of the Brazilian isolates occupied an intermediate node part way between the Brazilian and Argentinean clusters perhaps indicating that similar processes are leading to endemic disease elsewhere. Information from Chilean isolates would provide useful additional information, as this is the most recent South American country to report the expansion of MenW:cc11 disease. One of the advantages of the PubMLST *Neisseria* database is that comparable data are available to reference laboratories worldwide and will facilitate the monitoring of global epidemiology.

Owing to the high case fatality rates and persistence of MenW:cc11 disease in South America, the relatedness of the currently expanding UK strain is of concern. The English/Welsh strain has already exhibited similar behaviour by expanding first among the elderly and subsequently all age groups. Results of the current study show that the strain was being carried in at least five of the six institutions partaking in the UK carriage study from 2010 to 2012.^23^ In June 2013 the UK introduced a serogroup C booster among adolescents to maintain herd protection in cohorts vaccinated in infancy/early childhood since the initial introduction of the vaccine.^28^ Concerns over the accelerated increase in endemic serogroup W disease caused by a particularly virulent strain have prompted plans to offer quadrivalent serogroup A, C, W and Y vaccine to teenagers later in 2015. The universal presence of full length *nadA* genes within this strain indicates that a recently licensed protein-based serogroup B vaccine Bexsero^29^ may afford protection when it is introduced for use in infants in September 2015^30^ and this is supported by early hSBA studies using pooled pre- and post-vaccination infant sera (unpublished data).

In summary, large-scale genomic comparisons of a broad panel of geographically and temporally varied isolates revealed a genetically diverse global cc11 population. There are numerous strains currently circulating in different countries, some of which exhibit different epidemiological properties. The comprehensive UK and South African panels and selected isolates representing various episodes such as the Hajj outbreak and South American MenW:cc11 isolates are likely to be highly representative of their respective countries/cc11 disease episodes. The numerous miscellaneous isolates, although not necessarily representative of their respective countries, provide a diverse geo-temporal backdrop with which to view these episodes whilst maintaining a population structure dominated by two main sublineages. As further isolates/genomes are added from different countries and times, the overall picture will develop further. The analyses described here are readily reproducible using pubMLST.org infrastructure and will facilitate ongoing high-resolution global surveillance as more countries embrace routine WGS analysis. It will also better inform intervention strategies on national and global levels.

## Declaration of interests

Dr. du Plessis reports grants from Medical Research Council, South Africa, during the conduct of the study; Dr. Vazquez reports grants from Sanofi Pasteur, during the conduct of the study; grants and personal fees from Novartis Vaccines & Diagnostics, personal fees from GSK, grants and personal fees from Sanofi Pasteur, personal fees from Pfizer, personal fees from Baxter, outside the submitted work; Dr. Jolley reports grants from Wellcome Trust, during the conduct of the study; other from Crucell, outside the submitted work; Prof. Maiden reports grants and personal fees from Novartis, outside the submitted work.

Dr. Lucidarme, Ms. Bratcher, Ms. Hill, Dr. Gray, Dr. Tsang, Dr. Taha, Dr. Efron, Dr. Gorla, Dr. Findlow and Prof. Borrow have nothing to disclose.
