# Supplementary material for: Genomic resolution of an aggressive, widespread, diverse and expanding meningococcal serogroup B, C and W lineage
Source: J Infect. 2015 Nov;71(5):544–52. doi: 10.1016/j.jinf.2015.07.007 (PMC4635312; doi:10.1016/j.jinf.2015.07.007)
Supplement: Supplementary file 1 [file mmc1.docx]

**Web extra material**

**Supplementary table 1 – List of ST-11 clonal complex isolates/genomes included in the study.**

| **ID** | **Isolate** | **Lineage** | **Comment** | **Country** | **Year** | **Serogroup** | **PorA_VR1** | **PorA_VR2** | **ST** |
| --- | --- | --- | --- | --- | --- | --- | --- | --- | --- |
| 29333 | 11924 | 11.1 | Endemic South African strain | South Africa | 2003 | W | 5 | 2 | 11 |
| 21578 | SA_serogroup W_NM6 | 11.1 | Endemic South African strain | South Africa | 2004 | W | 5 | 2 | 11 |
| 29316 | 13283 | 11.1 | Endemic South African strain | South Africa | 2004 | W | 5 | 2 | 11 |
| 29406 | 15823 | 11.1 | Endemic South African strain | South Africa | 2004 | W | 5 | 2 | 11 |
| 21588 | SA_serogroup W_NM16 | 11.1 | Endemic South African strain | South Africa | 2005 | W | 5 | 2 | 11 |
| 29326 | 2266 | 11.1 | Endemic South African strain | South Africa | 2005 | W | 5 | 2 | 11 |
| 29337 | 1356 | 11.1 | Endemic South African strain | South Africa | 2005 | W | 5 | 2 | 11 |
| 29370 | 5032 | 11.1 | Endemic South African strain | South Africa | 2005 | W | 5 | 2 | 11 |
| 29371 | 2725 | 11.1 | Endemic South African strain | South Africa | 2005 | W | 5 | 2 | 11 |
| 29382 | 3390 | 11.1 | Endemic South African strain | South Africa | 2005 | W | 5 | 2 | 11 |
| 29384 | 4844 | 11.1 | Endemic South African strain | South Africa | 2005 | W | 5 | 2 | 11 |
| 29401 | 3308 | 11.1 | Endemic South African strain | South Africa | 2005 | W | 5 | 2 | 11 |
| 29422 | 3025 | 11.1 | Endemic South African strain | South Africa | 2005 | W | 5 | 2 | 11 |
| 21584 | SA_serogroup W_NM12 | 11.1 | Endemic South African strain | South Africa | 2006 | W | 5 | 2 | 11 |
| 29320 | 5453 | 11.1 | Endemic South African strain | South Africa | 2006 | W | 5 | 2 | 11 |
| 29338 | 8881 | 11.1 | Endemic South African strain | South Africa | 2006 | W | 5 | 2 | 11 |
| 29351 | 5855 | 11.1 | Endemic South African strain | South Africa | 2006 | W | 5 | 2 | 11 |
| 29374 | 7764 | 11.1 | Endemic South African strain | South Africa | 2006 | W | 5 | 2 | 11 |
| 29397 | 7313 | 11.1 | Endemic South African strain | South Africa | 2006 | W | 5 | 2 | 11 |
| 29421 | 8373 | 11.1 | Endemic South African strain | South Africa | 2006 | W | 5 | 2 | 11 |
| 29427 | 7789 | 11.1 | Endemic South African strain | South Africa | 2006 | W | 5 | 2 | 11 |
| 29437 | 6993 | 11.1 | Endemic South African strain | South Africa | 2006 | W | 5 | 2 | 11 |
| 29321 | 14493 | 11.1 | Endemic South African strain | South Africa | 2007 | W | 5 | 2 | 11 |
| 29322 | 12198 | 11.1 | Endemic South African strain | South Africa | 2007 | W | 5 | 2 | 11 |
| 29327 | 14983 | 11.1 | Endemic South African strain | South Africa | 2007 | W | 5 | 2 | 11 |
| 29342 | 15736 | 11.1 | Endemic South African strain | South Africa | 2007 | W | 5 | 2 | 11 |
| 29357 | 15279 | 11.1 | Endemic South African strain | South Africa | 2007 | W | 5 | 2 | 11 |
| 29375 | 15766 | 11.1 | Endemic South African strain | South Africa | 2007 | W | 5 | 2 | 11 |
| 29377 | 14395 | 11.1 | Endemic South African strain | South Africa | 2007 | W | 5 | 2 | 11 |
| 29419 | 13719 | 11.1 | Endemic South African strain | South Africa | 2007 | W | 5 | 2 | 11 |
| 29424 | 15663 | 11.1 | Endemic South African strain | South Africa | 2007 | W | 5 | 2 | 11 |
| 29438 | 14301 | 11.1 | Endemic South African strain | South Africa | 2007 | W | 5 | 2 | 4977 |
| 21573 | SA_serogroup W_NM1 | 11.1 | Endemic South African strain | South Africa | 2008 | W | 5 | 2 | 11 |
| 29317 | 20033 | 11.1 | Endemic South African strain | South Africa | 2008 | W | 5 | 2 | 11 |
| 29359 | 18470 | 11.1 | Endemic South African strain | South Africa | 2008 | W | 5 | 2 | 11 |
| 29383 | 16486 | 11.1 | Endemic South African strain | South Africa | 2008 | W | 5 | 2 | 11 |
| 29393 | 19208 | 11.1 | Endemic South African strain | South Africa | 2008 | W | 5 | 2 | 11 |
| 29395 | 17195 | 11.1 | Endemic South African strain | South Africa | 2008 | W | 5 | 2 | 11 |
| 29410 | 15783 | 11.1 | Endemic South African strain | South Africa | 2008 | W | 5 | 2 | 11 |
| 29415 | 17561 | 11.1 | Endemic South African strain | South Africa | 2008 | W | 5 | 2 | 11 |
| 29416 | 19923 | 11.1 | Endemic South African strain | South Africa | 2008 | W | 5 | 2 | 11 |
| 29339 | 21313 | 11.1 | Endemic South African strain | South Africa | 2009 | W | 5 | 2 | 11 |
| 29347 | 24836 | 11.1 | Endemic South African strain | South Africa | 2009 | W | 5 | 2 | 11 |
| 29348 | 25348 | 11.1 | Endemic South African strain | South Africa | 2009 | W | 5 | 2 | 11 |
| 29355 | 25472 | 11.1 | Endemic South African strain | South Africa | 2009 | W | 5 | 2 | 11 |
| 29356 | 21974 | 11.1 | Endemic South African strain | South Africa | 2009 | W | 5 | 2 | 11 |
| 29367 | 24204 | 11.1 | Endemic South African strain | South Africa | 2009 | W | 5 | 2 | 11 |
| 29408 | 21822 | 11.1 | Endemic South African strain | South Africa | 2009 | W | 5 | 2 | 11 |
| 29435 | 22774 | 11.1 | Endemic South African strain | South Africa | 2009 | W | 5 | 2 | 11 |
| 21582 | SA_serogroup W_NM10 | 11.1 | Endemic South African strain | South Africa | 2010 | W | 5 | 2 | 11 |
| 29332 | 30147 | 11.1 | Endemic South African strain | South Africa | 2010 | W | 5 | 2 | 11 |
| 29336 | 29572 | 11.1 | Endemic South African strain | South Africa | 2010 | W | 5 | 2 | 11 |
| 29386 | 28517 | 11.1 | Endemic South African strain | South Africa | 2010 | W | 5 | 2 | 11 |
| 29388 | 30494 | 11.1 | Endemic South African strain | South Africa | 2010 | W | 5 | 2 | 11 |
| 29412 | 28319 | 11.1 | Endemic South African strain | South Africa | 2010 | W | 5 | 2 | 11 |
| 29414 | 28189 | 11.1 | Endemic South African strain | South Africa | 2010 | W | 5 | 2 | 1287 |
| 29417 | 29246 | 11.1 | Endemic South African strain | South Africa | 2010 | W | 5 | 2 | 11 |
| 29425 | 26790 | 11.1 | Endemic South African strain | South Africa | 2010 | W | 5 | 2 | 11 |
| 21583 | SA_serogroup W_NM11 | 11.1 | Endemic South African strain | South Africa | 2011 | W | 5 | 2 | 11 |
| 29361 | 33281 | 11.1 | Endemic South African strain | South Africa | 2011 | W | 5 | 2 | 11 |
| 29368 | 31847 | 11.1 | Endemic South African strain | South Africa | 2011 | W | 5 | 2 | 11 |
| 29400 | 34047 | 11.1 | Endemic South African strain | South Africa | 2011 | W | 5 | 2 | 11 |
| 29418 | 34455 | 11.1 | Endemic South African strain | South Africa | 2011 | W | 5 | 2 | 11 |
| 29428 | 33702 | 11.1 | Endemic South African strain | South Africa | 2011 | W | 5 | 2 | 11 |
| 29323 | 37252 | 11.1 | Endemic South African strain | South Africa | 2012 | W | 5 | 2 | 11 |
| 29346 | 35813 | 11.1 | Endemic South African strain | South Africa | 2012 | W | 5 | 2 | 11 |
| 29360 | 35487 | 11.1 | Endemic South African strain | South Africa | 2012 | W | 5 | 2 | 11 |
| 29369 | 34873 | 11.1 | Endemic South African strain | South Africa | 2012 | W | 5 | 2 | 11 |
| 29373 | 36146 | 11.1 | Endemic South African strain | South Africa | 2012 | W | 5 | 2 | 11 |
| 29389 | 35371 | 11.1 | Endemic South African strain | South Africa | 2012 | W | 5 | 2 | 11 |
| 29405 | 36527 | 11.1 | Endemic South African strain | South Africa | 2012 | W | 5 | 2 | 11 |
| 29313 | 39319 | 11.1 | Endemic South African strain | South Africa | 2013 | W | 5 | 2 | 11 |
| 29314 | 39619 | 11.1 | Endemic South African strain | South Africa | 2013 | W | 5 | 2 | 11 |
| 29318 | 40328 | 11.1 | Endemic South African strain | South Africa | 2013 | W | 5 | 2 | 11 |
| 29387 | 41003 | 11.1 | Endemic South African strain | South Africa | 2013 | W | 5 | 2 | 11 |
| 29396 | 40559 | 11.1 | Endemic South African strain | South Africa | 2013 | W | 5 | 2 | 11 |
| 29402 | 40498 | 11.1 | Endemic South African strain | South Africa | 2013 | W | 5 | 2 | 11 |
| 29707 | M07 240118 | 11.1 | Endemic South African strain | UK | 2007 | W | 5 | 2 | 11 |
| 20424 | M11 240389 | 11.1 | Endemic South African strain | UK | 2011 | W | 5 | 2 | 11 |
|  |  |  |  |  |  |  |  |  |  |
| 30080 | 2001076 | 11.1 | Anglo-French Hajj strain | Algeria | 2001 | W | 5 | 2 | 11 |
| 30081 | 2002015 | 11.1 | Anglo-French Hajj strain | Cameroon | 2001 | W | 5 | 2 | 11 |
| 30082 | 2002016 | 11.1 | Anglo-French Hajj strain | Cameroon | 2001 | W | 5 | 2 | 11 |
| 30085 | 2002029 | 11.1 | Anglo-French Hajj strain | Cameroon | 2001 | W | 5 | 2 | 11 |
| 30086 | 2001068 | 11.1 | Anglo-French Hajj strain | Chad | 2001 | W | 5 | 2 | 11 |
| 30091 | 2002058 | 11.1 | Anglo-French Hajj strain | Niger | 2002 | W | 5 | 2 | 11 |
| 30075 | 2000081 | 11.1 | Anglo-French Hajj strain | Senegal | 2000 | W | 5 | 2 | 11 |
| 30079 | 2000058 | 11.1 | Anglo-French Hajj strain | Senegal | 2000 | W | 5 | 2 | 11 |
| 30107 | 2001069 | 11.1 | Anglo-French Hajj strain | Senegal | 2001 | W | 5 | 2 | 11 |
| 30066 | M14 240068 | 11.1 | Anglo-French Hajj strain | Turkey | 2005 | W | 5 | 2 | 11 |
| 30065 | M14 240067 | 11.1 | Anglo-French Hajj strain | Turkey | 2006 | W | 5 | 2 | 11 |
| 30067 | M14 240069 | 11.1 | Anglo-French Hajj strain | Turkey | 2006 | W | 5 | 2 | 11 |
| 19957 | M00 241317 | 11.1 | Anglo-French Hajj strain | UK | 2000 | W | 5 | 2 | 11 |
| 29775 | M00 241395 | 11.1 | Anglo-French Hajj strain | UK | 2000 | W | 5 | 2 | 11 |
| 29928 | M00 241341 | 11.1 | Anglo-French Hajj strain | UK | 2000 | W | 5 | 2 | 11 |
| 29929 | M00 241352 | 11.1 | Anglo-French Hajj strain | UK | 2000 | W | 5 | 2 | 11 |
| 29930 | M00 241357 | 11.1 | Anglo-French Hajj strain | UK | 2000 | W | 5 | 2 | 11 |
| 29931 | M00 241362 | 11.1 | Anglo-French Hajj strain | UK | 2000 | W | 5 | 2 | 11 |
| 29932 | M00 241380 | 11.1 | Anglo-French Hajj strain | UK | 2000 | W | 5 | 2 | 11 |
| 29933 | M00 241396 | 11.1 | Anglo-French Hajj strain | UK | 2000 | W | 5 | 2 | 11 |
| 29934 | M00 241401 | 11.1 | Anglo-French Hajj strain | UK | 2000 | W | 5 | 2 | 11 |
| 29935 | M00 241408 | 11.1 | Anglo-French Hajj strain | UK | 2000 | W | 5 | 2 | 11 |
| 29936 | M00 241420 | 11.1 | Anglo-French Hajj strain | UK | 2000 | W | 5 | 2 | 11 |
| 29937 | M00 241430 | 11.1 | Anglo-French Hajj strain | UK | 2000 | W | 5 | 2 | 11 |
| 29938 | M00 241431 | 11.1 | Anglo-French Hajj strain | UK | 2000 | W | 5 | 2 | 11 |
| 29939 | M00 241432 | 11.1 | Anglo-French Hajj strain | UK | 2000 | W | 5 | 2 | 11 |
| 29941 | M00 241435 | 11.1 | Anglo-French Hajj strain | UK | 2000 | W | 5 | 2 | 11 |
| 29942 | M00 241445 | 11.1 | Anglo-French Hajj strain | UK | 2000 | W | 5 | 2 | 11 |
| 29943 | M00 241447 | 11.1 | Anglo-French Hajj strain | UK | 2000 | W | 5 | 2 | 11 |
| 29944 | M00 241491 | 11.1 | Anglo-French Hajj strain | UK | 2000 | W | 5 | 2 | 11 |
| 29945 | M00 241510 | 11.1 | Anglo-French Hajj strain | UK | 2000 | W | 5 | 2 | 11 |
| 29946 | M00 241511 | 11.1 | Anglo-French Hajj strain | UK | 2000 | W | 5 | 2 | 11 |
| 29947 | M00 241557 | 11.1 | Anglo-French Hajj strain | UK | 2000 | W | 5 | 2 | 11 |
| 29948 | M00 241578 | 11.1 | Anglo-French Hajj strain | UK | 2000 | W | 5 | 2 | 11 |
| 29949 | M00 241620 | 11.1 | Anglo-French Hajj strain | UK | 2000 | W | 5 | 2 | 11 |
| 29950 | M00 241645 | 11.1 | Anglo-French Hajj strain | UK | 2000 | W | 5 | 2 | 11 |
| 29951 | M00 241655 | 11.1 | Anglo-French Hajj strain | UK | 2000 | W | 5 | 2 | 11 |
| 29953 | M00 241674 | 11.1 | Anglo-French Hajj strain | UK | 2000 | W | 5 | 2 | 11 |
| 29954 | M00 241675 | 11.1 | Anglo-French Hajj strain | UK | 2000 | W | 5 | 2 | 11 |
| 29955 | M00 241704 | 11.1 | Anglo-French Hajj strain | UK | 2000 | W | 5 | 2 | 11 |
| 29956 | M00 241759 | 11.1 | Anglo-French Hajj strain | UK | 2000 | W | 5 | 2 | 11 |
| 29957 | M00 241760 | 11.1 | Anglo-French Hajj strain | UK | 2000 | W | 5 | 2 | 11 |
| 29958 | M00 241800 | 11.1 | Anglo-French Hajj strain | UK | 2000 | W | 5 | 2 | 11 |
| 29959 | M00 241833 | 11.1 | Anglo-French Hajj strain | UK | 2000 | W | 5 | 2 | 11 |
| 29960 | M00 241894 | 11.1 | Anglo-French Hajj strain | UK | 2000 | W | 5 | 2 | 11 |
| 29961 | M00 241957 | 11.1 | Anglo-French Hajj strain | UK | 2000 | W | 5 | 2 | 11 |
| 29962 | M00 241978 | 11.1 | Anglo-French Hajj strain | UK | 2000 | W | 5 | 2 | 11 |
| 29963 | M00 241986 | 11.1 | Anglo-French Hajj strain | UK | 2000 | W | 5 | 2 | 11 |
| 29964 | M00 242029 | 11.1 | Anglo-French Hajj strain | UK | 2000 | W | 5 | 2 | 11 |
| 29965 | M00 242295 | 11.1 | Anglo-French Hajj strain | UK | 2000 | W | 5 | 2 | 11 |
| 29966 | M00 242531 | 11.1 | Anglo-French Hajj strain | UK | 2000 | W | 5 | 2 | 11 |
| 29967 | M00 242667 | 11.1 | Anglo-French Hajj strain | UK | 2000 | W | 5 | 2 | 11 |
| 29968 | M00 242770 | 11.1 | Anglo-French Hajj strain | UK | 2000 | W | 5 | 2 | 11 |
| 29969 | M00 242916 | 11.1 | Anglo-French Hajj strain | UK | 2000 | W | 5 | 2 | 11 |
| 29970 | M00 242974 | 11.1 | Anglo-French Hajj strain | UK | 2000 | W | 5 | 2 | 11 |
| 29991 | M00 243175 | 11.1 | Anglo-French Hajj strain | UK | 2000 | W | 5 | 2 | 11 |
| 29992 | M00 243297 | 11.1 | Anglo-French Hajj strain | UK | 2000 | W | 5 | 2 | 11 |
| 30122 | M00 241434 | 11.1 | Anglo-French Hajj strain | UK | 2000 | W | 5 | 2 | 11 |
| 30123 | M00 241979 | 11.1 | Anglo-French Hajj strain | UK | 2000 | W | 5 | 2 | 11 |
| 29677 | M01 242608 | 11.1 | Anglo-French Hajj strain | UK | 2001 | W | 5 | 2 | 11 |
| 29680 | M01 242591 | 11.1 | Anglo-French Hajj strain | UK | 2001 | W | 5 | 2 | 11 |
| 29681 | M01 242700 | 11.1 | Anglo-French Hajj strain | UK | 2001 | W | 5 | 2 | 11 |
| 29684 | M01 242717 | 11.1 | Anglo-French Hajj strain | UK | 2001 | W | 5 | 2 | 11 |
| 29994 | M01 240028 | 11.1 | Anglo-French Hajj strain | UK | 2001 | W | 5 | 2 | 11 |
| 29995 | M01 240168 | 11.1 | Anglo-French Hajj strain | UK | 2001 | W | 5 | 2 | 11 |
| 29996 | M01 240240 | 11.1 | Anglo-French Hajj strain | UK | 2001 | W | 5 | 2 | 11 |
| 29997 | M01 240241 | 11.1 | Anglo-French Hajj strain | UK | 2001 | W | 5 | 2 | 11 |
| 29998 | M01 240244 | 11.1 | Anglo-French Hajj strain | UK | 2001 | W | 5 | 2 | 11 |
| 29999 | M01 240354 | 11.1 | Anglo-French Hajj strain | UK | 2001 | W | 5 | 2 | 11 |
| 30001 | M01 240634 | 11.1 | Anglo-French Hajj strain | UK | 2001 | W | 5 | 2 | 11 |
| 30002 | M01 240659 | 11.1 | Anglo-French Hajj strain | UK | 2001 | W | 5 | 2 | 11 |
| 30004 | M01 240759 | 11.1 | Anglo-French Hajj strain | UK | 2001 | W | 5 | 2 | 11 |
| 30005 | M01 240760 | 11.1 | Anglo-French Hajj strain | UK | 2001 | W | 5 | 2 | 11 |
| 30006 | M01 240801 | 11.1 | Anglo-French Hajj strain | UK | 2001 | W | 5 | 2 | 11 |
| 30007 | M01 240807 | 11.1 | Anglo-French Hajj strain | UK | 2001 | W | 5 | 2 | 11 |
| 30008 | M01 240808 | 11.1 | Anglo-French Hajj strain | UK | 2001 | W | 5 | 2 | 11 |
| 30009 | M01 240839 | 11.1 | Anglo-French Hajj strain | UK | 2001 | W | 5 | 2 | 11 |
| 30010 | M01 240840 | 11.1 | Anglo-French Hajj strain | UK | 2001 | W | 5 | 2 | 11 |
| 30011 | M01 240859 | 11.1 | Anglo-French Hajj strain | UK | 2001 | W | 5 | 2 | 11 |
| 30012 | M01 240887 | 11.1 | Anglo-French Hajj strain | UK | 2001 | W | 5 | 2 | 11 |
| 30013 | M01 240900 | 11.1 | Anglo-French Hajj strain | UK | 2001 | W | 5 | 2 | 11 |
| 30014 | M01 240902 | 11.1 | Anglo-French Hajj strain | UK | 2001 | W | 5 | 2 | 11 |
| 30015 | M01 240913 | 11.1 | Anglo-French Hajj strain | UK | 2001 | W | 5 | 2 | 11 |
| 30016 | M01 240915 | 11.1 | Anglo-French Hajj strain | UK | 2001 | W | 5 | 2 | 11 |
| 30017 | M01 240916 | 11.1 | Anglo-French Hajj strain | UK | 2001 | W | 5 | 2 | 11 |
| 30018 | M01 240921 | 11.1 | Anglo-French Hajj strain | UK | 2001 | W | 5 | 2 | 11 |
| 30019 | M01 240949 | 11.1 | Anglo-French Hajj strain | UK | 2001 | W | 5 | 2 | 11 |
| 30020 | M01 240953 | 11.1 | Anglo-French Hajj strain | UK | 2001 | W | 5 | 2 | 11 |
| 30021 | M01 240956 | 11.1 | Anglo-French Hajj strain | UK | 2001 | W | 5 | 2 | 11 |
| 30022 | M01 240959 | 11.1 | Anglo-French Hajj strain | UK | 2001 | W | 5 | 2 | 11 |
| 30023 | M01 240967 | 11.1 | Anglo-French Hajj strain | UK | 2001 | W | 5 | 2 | 11 |
| 30024 | M01 240978 | 11.1 | Anglo-French Hajj strain | UK | 2001 | W | 5 | 2 | 11 |
| 30025 | M01 240979 | 11.1 | Anglo-French Hajj strain | UK | 2001 | W | 5 | 2 | 11 |
| 30026 | M01 240989 | 11.1 | Anglo-French Hajj strain | UK | 2001 | W | 5 | 2 | 11 |
| 30027 | M01 240990 | 11.1 | Anglo-French Hajj strain | UK | 2001 | W | 5 | 2 | 11 |
| 30028 | M01 241031 | 11.1 | Anglo-French Hajj strain | UK | 2001 | W | 5 | 2 | 11 |
| 30029 | M01 241052 | 11.1 | Anglo-French Hajj strain | UK | 2001 | W | 5 | 2 | 11 |
| 30030 | M01 241064 | 11.1 | Anglo-French Hajj strain | UK | 2001 | W | 5 | 2 | 11 |
| 30031 | M01 241093 | 11.1 | Anglo-French Hajj strain | UK | 2001 | W | 5 | 2 | 11 |
| 30032 | M01 241097 | 11.1 | Anglo-French Hajj strain | UK | 2001 | W | 5 | 2 | 11 |
| 30033 | M01 241098 | 11.1 | Anglo-French Hajj strain | UK | 2001 | W | 5 | 2 | 11 |
| 30034 | M01 241133 | 11.1 | Anglo-French Hajj strain | UK | 2001 | W | 5 | 2 | 11 |
| 30035 | M01 241213 | 11.1 | Anglo-French Hajj strain | UK | 2001 | W | 5 | 2 | 11 |
| 30036 | M01 241214 | 11.1 | Anglo-French Hajj strain | UK | 2001 | W | 5 | 2 | 11 |
| 30037 | M01 241368 | 11.1 | Anglo-French Hajj strain | UK | 2001 | W | 5 | 2 | 11 |
| 30038 | M01 241396 | 11.1 | Anglo-French Hajj strain | UK | 2001 | W | 5 | 2 | 11 |
| 30039 | M01 241454 | 11.1 | Anglo-French Hajj strain | UK | 2001 | W | 5 | 2 | 11 |
| 30040 | M01 241494 | 11.1 | Anglo-French Hajj strain | UK | 2001 | W | 5 | 2 | 11 |
| 30041 | M01 241950 | 11.1 | Anglo-French Hajj strain | UK | 2001 | W | 5 | 2 | 11 |
| 30043 | M01 242084 | 11.1 | Anglo-French Hajj strain | UK | 2001 | W | 5 | 2 | 11 |
| 30265 | M01 242103 | 11.1 | Anglo-French Hajj strain | UK | 2001 | W | 5 | 2 | 11 |
| 30266 | M01 242502 | 11.1 | Anglo-French Hajj strain | UK | 2001 | W | 5 | 2 | 11 |
| 30267 | M01 242503 | 11.1 | Anglo-French Hajj strain | UK | 2001 | W | 5 | 2 | 11 |
| 29683 | M02 240124 | 11.1 | Anglo-French Hajj strain | UK | 2002 | W | 5 | 2 | 11 |
| 29686 | M02 240227 | 11.1 | Anglo-French Hajj strain | UK | 2002 | W | 5 | 2 | 11 |
| 29688 | M02 240422 | 11.1 | Anglo-French Hajj strain | UK | 2002 | W | 5 | 2 | 11 |
| 29689 | M02 240440 | 11.1 | Anglo-French Hajj strain | UK | 2002 | W | 5 | 2 | 11 |
| 29690 | M02 240609 | 11.1 | Anglo-French Hajj strain | UK | 2002 | W | 5 | 2 | 11 |
| 29692 | M02 240839 | 11.1 | Anglo-French Hajj strain | UK | 2002 | W | 5 | 2 | 11 |
| 29694 | M02 241321 | 11.1 | Anglo-French Hajj strain | UK | 2002 | W | 5 | 2 | 11 |
| 29695 | M02 241603 | 11.1 | Anglo-French Hajj strain | UK | 2002 | W | 5 | 2 | 11 |
| 29696 | M02 241666 | 11.1 | Anglo-French Hajj strain | UK | 2002 | W | 5 | 2 | 11 |
| 29697 | M02 241746 | 11.1 | Anglo-French Hajj strain | UK | 2002 | W | 5 | 2 | 11 |
| 29699 | M03 240692 | 11.1 | Anglo-French Hajj strain | UK | 2003 | W | 5 | 2 | 11 |
| 29700 | M03 240696 | 11.1 | Anglo-French Hajj strain | UK | 2003 | W | 5 | 2 | 11 |
| 29702 | M04 240429 | 11.1 | Anglo-French Hajj strain | UK | 2004 | W | 5 | 2 | 11 |
| 29703 | M04 240435 | 11.1 | Anglo-French Hajj strain | UK | 2004 | W | 5 | 2 | 11 |
| 31164 | M14 240443 | 11.1 | Anglo-French Hajj strain | France | 2000 | W | 5 | 2 | 11 |
| 31165 | M14 240444 | 11.1 | Anglo-French Hajj strain | France | 2000 | W | 5 | 2 | 11 |
| 31167 | M14 240446 | 11.1 | Anglo-French Hajj strain | France | 2014 | W | 5 | 2 | 11 |
|  |  |  |  |  |  |  |  |  |  |
| 30106 | 2001215 | 11.1 | Burkina Faso/North African isolates | Burkina Faso | 2001 | W | 5 | 2 | 11 |
| 30098 | 2004264 | 11.1 | Burkina Faso/North African isolates | Burkina Faso | 2004 | W | 5 | 2 | 11 |
| 30099 | 2004265 | 11.1 | Burkina Faso/North African isolates | Burkina Faso | 2004 | W | 5 | 2 | 11 |
| 30101 | 2004268 | 11.1 | Burkina Faso/North African isolates | Burkina Faso | 2004 | W | 5 | 2 | 11 |
| 30087 | 2002018 | 11.1 | Burkina Faso/North African isolates | Niger | 2001 | W | 5 | 2 | 11 |
| 30088 | 2002019 | 11.1 | Burkina Faso/North African isolates | Niger | 2001 | W | 5 | 2 | 11 |
| 30089 | 2002039 | 11.1 | Burkina Faso/North African isolates | Niger | 2002 | W | 5 | 2 | 11 |
| 30090 | 2002040 | 11.1 | Burkina Faso/North African isolates | Niger | 2002 | W | 5 | 2 | 11 |
| 30092 | 2002059 | 11.1 | Burkina Faso/North African isolates | Niger | 2002 | W | 5 | 2 | 11 |
| 30093 | 2003019 | 11.1 | Burkina Faso/North African isolates | Niger | 2003 | W | 5 | 2 | 11 |
| 30094 | 2003034 | 11.1 | Burkina Faso/North African isolates | Niger | 2003 | W | 5 | 2 | 11 |
| 30095 | 2003035 | 11.1 | Burkina Faso/North African isolates | Niger | 2003 | W | 5 | 2 | 11 |
| 30096 | 2003054 | 11.1 | Burkina Faso/North African isolates | Niger | 2003 | W | 5 | 2 | 11 |
| 30097 | 2003055 | 11.1 | Burkina Faso/North African isolates | Niger | 2003 | W | 5 | 2 | 11 |
| 30180 | M98 251534 | 11.1 | Burkina Faso/North African isolates | UK | 1998 | W | 5 | 2 | 11 |
|  |  |  |  |  |  |  |  |  |  |
| 30104 | 2001213 | 11.1 | Burkina Faso/North African isolates | Burkina Faso | 2001 | W | 5 | 2 | 11 |
| 30105 | 2001214 | 11.1 | Burkina Faso/North African isolates | Burkina Faso | 2001 | W | 5 | 2 | 11 |
| 27087 | LNP19995 | 11.1 | Burkina Faso/North African isolates | Burkina Faso | 2002 | W | 5 | 2 | 11 |
| 30100 | 2004267 | 11.1 | Burkina Faso/North African isolates | Burkina Faso | 2004 | W | 5 | 2 | 11 |
| 30076 | 2000175 | 11.1 | Burkina Faso/North African isolates | Cameroon | 2000 | W | 5 | 2 | 11 |
| 30077 | 2000176 | 11.1 | Burkina Faso/North African isolates | Cameroon | 2000 | W | 5 | 2 | 11 |
| 30078 | 2002011 | 11.1 | Burkina Faso/North African isolates | Cameroon | 2000 | W | 5 | 2 | 11 |
| 30083 | 2002021 | 11.1 | Burkina Faso/North African isolates | Cameroon | 2001 | W | 5 | 2 | 11 |
| 29679 | M01 242679 | 11.1 | Burkina Faso/North African isolates | UK | 2001 | W | 5 | 2 | 11 |
| 29705 | M06 240459 | 11.1 | Burkina Faso/North African isolates | UK | 2006 | W | 5 | 2 | 11 |
|  |  |  |  |  |  |  |  |  |  |
| 30074 | 2001001 | 11.1 | lineage 11.1 distal region, other | Algeria | 1999 | W | 5 | 2 | 11 |
| 30073 | 96057 | 11.1 | lineage 11.1 distal region, other | Chad | 1996 | W | 5 | 2 | 11 |
| 21587 | SA_serogroup W_NM15 | 11.1 | lineage 11.1 distal region, other | South Africa | 2003 | W | 5 | 2 | 11 |
| 29398 | 10093 | 11.1 | lineage 11.1 distal region, other | South Africa | 2003 | W | 5 | 2 | 11 |
| 29439 | 10803 | 11.1 | lineage 11.1 distal region, other | South Africa | 2003 | W | 5 | 2 | 11 |
| 29315 | 16661 | 11.1 | lineage 11.1 distal region, other | South Africa | 2004 | W | 5 | 2 | 11 |
| 29329 | 17690 | 11.1 | lineage 11.1 distal region, other | South Africa | 2004 | W | 5 | 2 | 11 |
| 29330 | 15984 | 11.1 | lineage 11.1 distal region, other | South Africa | 2004 | W | 5 | 2 | 11 |
| 29381 | 16187 | 11.1 | lineage 11.1 distal region, other | South Africa | 2004 | W | 5 | 2 | 11 |
| 30235 | M96 253521 | 11.1 | lineage 11.1 distal region, other | UK | 1996 | W | 5 | 2 | 11 |
| 30236 | M96 255203 | 11.1 | lineage 11.1 distal region, other | UK | 1996 | W | 5 | 2 | 11 |
| 30238 | M96 256246 | 11.1 | lineage 11.1 distal region, other | UK | 1996 | W | 5 | 2 | 11 |
| 30174 | M97 250455 | 11.1 | lineage 11.1 distal region, other | UK | 1997 | W | 5 | 2 | 11 |
| 30176 | M97 251635 | 11.1 | lineage 11.1 distal region, other | UK | 1997 | W | 5 | 2 | 11 |
| 30179 | M98 251039 | 11.1 | lineage 11.1 distal region, other | UK | 1998 | W | 5 | 2 | 11 |
| 30181 | M98 253527 | 11.1 | lineage 11.1 distal region, other | UK | 1998 | W | 5 | 2 | 11 |
| 29952 | M00 241673 | 11.1 | lineage 11.1 distal region, other | UK | 2000 | W | 5 | 2 | 11 |
| 29411 | 16421 | 11.1 | lineage 11.1 distal region, other | South Africa | 2004 | W | 5 | 2 | 11 |
|  |  |  |  |  |  |  |  |  |  |
| 30183 | M99 242612 | 11.1 | lineage 11.1 distal region, other | Malta | 1999 | W | 5 | 2 | 11 |
| 30184 | M99 243856 | 11.1 | lineage 11.1 distal region, other | Malta | 1999 | W | 5 | 2 | 11 |
| 29704 | M05 241067 | 11.1 | lineage 11.1 distal region, other | UK | 2005 | W | 5 | 2-43 | 11 |
|  |  |  |  |  |  |  |  |  |  |
| 26898 | 12002_2013 | 11.1 | South American/UK strain | Ireland | 2013 | W | 5 | 2 | 11 |
| 26899 | 12003_2013 | 11.1 | South American/UK strain | Ireland | 2013 | W | 5 | 2 | 11 |
| 26914 | 12018_2013 | 11.1 | South American/UK strain | Ireland | 2013 | W | 5 | 2 | 11 |
| 29715 | M09 240862 | 11.1 | South American/UK strain | UK | 2009 | W | 5 | 2 | 11 |
| 29716 | M09 240900 | 11.1 | South American/UK strain | UK | 2009 | W | 5 | 2 | 11 |
| 19968 | M10 240514 | 11.1 | South American/UK strain | UK | 2010 | W | 5 | 2 | 11 |
| 20154 | M10 240817 | 11.1 | South American/UK strain | UK | 2010 | W | 5 | 2 | 11 |
| 20158 | M10 240821 | 11.1 | South American/UK strain | UK | 2010 | W | 5 | 2 | 11 |
| 29714 | M10 240005 | 11.1 | South American/UK strain | UK | 2010 | W | 5 | 2 | 11 |
| 29718 | M10 240389 | 11.1 | South American/UK strain | UK | 2010 | W | 5 | 2 | 11 |
| 29719 | M10 240452 | 11.1 | South American/UK strain | UK | 2010 | W | 5 | 2 | 11 |
| 29720 | M10 240696 | 11.1 | South American/UK strain | UK | 2010 | W | 5 | 2 | 11 |
| 20196 | M11 240035 | 11.1 | South American/UK strain | UK | 2011 | W | 5 | 2 | 11 |
| 20216 | M11 240057 | 11.1 | South American/UK strain | UK | 2011 | W | 5 | 2 | 11 |
| 20226 | M11 240067 | 11.1 | South American/UK strain | UK | 2011 | W | 5 | 2 | 11 |
| 20247 | M11 240099 | 11.1 | South American/UK strain | UK | 2011 | W | 5 | 2 | 11 |
| 20288 | M11 240168 | 11.1 | South American/UK strain | UK | 2011 | W | 5 | 2 | 11 |
| 20368 | M11 240305 | 11.1 | South American/UK strain | UK | 2011 | W | 5 | 2 | 11 |
| 20436 | M11 240403 | 11.1 | South American/UK strain | UK | 2011 | W | 5 | 2 | 11 |
| 20444 | M11 240417 | 11.1 | South American/UK strain | UK | 2011 | W | 5 | 2 | 11 |
| 20449 | M11 240427 | 11.1 | South American/UK strain | UK | 2011 | W | 5 | 2 | 11 |
| 21123 | M11 240486 | 11.1 | South American/UK strain | UK | 2011 | W | 5 | 2 | 1860 |
| 21163 | M11 240726 | 11.1 | South American/UK strain | UK | 2011 | W | 5 | 2 | 11 |
| 21203 | M11 240798 | 11.1 | South American/UK strain | UK | 2011 | W | 5 | 2 | 11 |
| 21206 | M11 240802 | 11.1 | South American/UK strain | UK | 2011 | W | 5 | 2 | 11 |
| 21214 | M11 240953 | 11.1 | South American/UK strain | UK | 2011 | W | 5 | 2 | 11 |
| 29813 | M11 240418 | 11.1 | South American/UK strain | UK | 2011 | W | 5 | 2 | 11 |
| 29814 | M11 240419 | 11.1 | South American/UK strain | UK | 2011 | W | 5 | 2 | 11 |
| 20460 | M12 240156 | 11.1 | South American/UK strain | UK | 2012 | W | 5 | 2 | 11 |
| 20461 | M12 240160 | 11.1 | South American/UK strain | UK | 2012 | W | 5 | 2 | 11 |
| 20462 | M12 240196 | 11.1 | South American/UK strain | UK | 2012 | W | 5 | 2 | 11 |
| 21288 | M12 240004 | 11.1 | South American/UK strain | UK | 2012 | W | 5 | 2 | 11 |
| 21298 | M12 240016 | 11.1 | South American/UK strain | UK | 2012 | W | 5 | 2 | 11 |
| 21302 | M12 240021 | 11.1 | South American/UK strain | UK | 2012 | W | 5 | 2 | 11 |
| 21305 | M12 240027 | 11.1 | South American/UK strain | UK | 2012 | W | 5 | 2 | 11 |
| 21334 | M12 240067 | 11.1 | South American/UK strain | UK | 2012 | W | 5 | 2 | 11 |
| 21354 | M12 240095 | 11.1 | South American/UK strain | UK | 2012 | W | 5 | 2 | 11 |
| 21375 | M12 240125 | 11.1 | South American/UK strain | UK | 2012 | W | 5 | 2 | 11 |
| 21377 | M12 240127 | 11.1 | South American/UK strain | UK | 2012 | W | 5 | 2 | 11 |
| 21381 | M12 240133 | 11.1 | South American/UK strain | UK | 2012 | W | 5 | 2 | 11 |
| 21386 | M12 240144 | 11.1 | South American/UK strain | UK | 2012 | W | 5 | 2 | 10284 |
| 21446 | M12 240240 | 11.1 | South American/UK strain | UK | 2012 | W | 5 | 2 | 11 |
| 21492 | M12 240317 | 11.1 | South American/UK strain | UK | 2012 | W | 5 | 2 | 11 |
| 21499 | M12 240324 | 11.1 | South American/UK strain | UK | 2012 | W | 5 | 2 | 11 |
| 28114 | M12 240337 | 11.1 | South American/UK strain | UK | 2012 | W | 5-1 | 10-4 | 11 |
| 28115 | M12 240640 | 11.1 | South American/UK strain | UK | 2012 | W | 5 | 2 | 11 |
| 28116 | M12 240657 | 11.1 | South American/UK strain | UK | 2012 | W | 5 | 2 | 11 |
| 28117 | M12 240663 | 11.1 | South American/UK strain | UK | 2012 | W | 7-2 | 14 | 11 |
| 28119 | M12 240702 | 11.1 | South American/UK strain | UK | 2012 | W | 5 | 2 | 11 |
| 28121 | M12 240751 | 11.1 | South American/UK strain | UK | 2012 | W | 5 | 2 | 10651 |
| 28122 | M12 240754 | 11.1 | South American/UK strain | UK | 2012 | W | 5 | 2 | 11 |
| 28125 | M12 240774 | 11.1 | South American/UK strain | UK | 2012 | W | 5 | 2 | 11 |
| 28128 | M12 240826 | 11.1 | South American/UK strain | UK | 2012 | W | 5 | 2 | 11 |
| 28131 | M12 240895 | 11.1 | South American/UK strain | UK | 2012 | W | 5 | 2 | 11 |
| 28132 | M12 240898 | 11.1 | South American/UK strain | UK | 2012 | W | 5 | 2 | 11 |
| 28134 | M13 240015 | 11.1 | South American/UK strain | UK | 2013 | W | 5 | 2 | 11 |
| 28135 | M13 240025 | 11.1 | South American/UK strain | UK | 2013 | W | 5 | 2 | 11 |
| 28136 | M13 240028 | 11.1 | South American/UK strain | UK | 2013 | W | 5 | 2 | 11 |
| 28137 | M13 240056 | 11.1 | South American/UK strain | UK | 2013 | W | 5 | 2 | 11 |
| 28138 | M13 240066 | 11.1 | South American/UK strain | UK | 2013 | W | 5 | 2 | 11 |
| 28139 | M13 240077 | 11.1 | South American/UK strain | UK | 2013 | W | 5 | 2 | 10651 |
| 28141 | M13 240109 | 11.1 | South American/UK strain | UK | 2013 | W | 5 | 2 | 11 |
| 28142 | M13 240114 | 11.1 | South American/UK strain | UK | 2013 | W | 5 | 2 | 11 |
| 28143 | M13 240158 | 11.1 | South American/UK strain | UK | 2013 | W | 5 | 2 | 11 |
| 28144 | M13 240168 | 11.1 | South American/UK strain | UK | 2013 | W | 5 | 2 | 11 |
| 28146 | M13 240185 | 11.1 | South American/UK strain | UK | 2013 | W | 5 | 2 | 10651 |
| 28147 | M13 240223 | 11.1 | South American/UK strain | UK | 2013 | W | 5 | 2 | 11 |
| 28148 | M13 240225 | 11.1 | South American/UK strain | UK | 2013 | W | 5 | 2 | 10651 |
| 28149 | M13 240238 | 11.1 | South American/UK strain | UK | 2013 | W | 5 | 2 | 11 |
| 28150 | M13 240246 | 11.1 | South American/UK strain | UK | 2013 | W | 5 | 2 | 11 |
| 28151 | M13 240247 | 11.1 | South American/UK strain | UK | 2013 | W | 5 | 2 | 11 |
| 28152 | M13 240251 | 11.1 | South American/UK strain | UK | 2013 | W | 5 | 2 | 11 |
| 28153 | M13 240269 | 11.1 | South American/UK strain | UK | 2013 | W | 5 | 2 | 11 |
| 28154 | M13 240283 | 11.1 | South American/UK strain | UK | 2013 | W | 5 | 2 | 11 |
| 28156 | M13 240436 | 11.1 | South American/UK strain | UK | 2013 | W | 5 | 2 | 11 |
| 28157 | M13 240446 | 11.1 | South American/UK strain | UK | 2013 | W | 5 | 2 | 11 |
| 28158 | M13 240457 | 11.1 | South American/UK strain | UK | 2013 | W | 5 | 2 | 11 |
| 28159 | M13 240464 | 11.1 | South American/UK strain | UK | 2013 | W | 21 | 16 | 11 |
| 28161 | M13 240467 | 11.1 | South American/UK strain | UK | 2013 | W | 5 | 2 | 11 |
| 28162 | M13 240469 | 11.1 | South American/UK strain | UK | 2013 | W | 5 | 2 | 11 |
| 28163 | M13 240473 | 11.1 | South American/UK strain | UK | 2013 | W | 5 | 2 | 11 |
| 28164 | M13 240482 | 11.1 | South American/UK strain | UK | 2013 | W | 5 | 2 | 11 |
| 29721 | M13 240491 | 11.1 | South American/UK strain | UK | 2013 | W | 5 | 2 | 11 |
| 29722 | M13 240530 | 11.1 | South American/UK strain | UK | 2013 | W | 5 | 2 | 11 |
| 29723 | M13 240555 | 11.1 | South American/UK strain | UK | 2013 | W | 5 | 2 | 11 |
| 29724 | M13 240591 | 11.1 | South American/UK strain | UK | 2013 | W | 5 | 2 | 11 |
| 29725 | M13 240603 | 11.1 | South American/UK strain | UK | 2013 | W | 5 | 2 | 11 |
| 29726 | M13 240604 | 11.1 | South American/UK strain | UK | 2013 | W | 5 | 2 | 10651 |
| 29727 | M13 240545 | 11.1 | South American/UK strain | UK | 2013 | W | 5 | 2 | 11 |
| 29728 | M13 240558 | 11.1 | South American/UK strain | UK | 2013 | W | 5 | 2 | 11 |
| 29729 | M13 240594 | 11.1 | South American/UK strain | UK | 2013 | W | 5 | 2 | 11 |
| 29731 | M13 240510 | 11.1 | South American/UK strain | UK | 2013 | W | 5 | 2 | 11 |
| 29732 | M13 240633 | 11.1 | South American/UK strain | UK | 2013 | W | 5 | 2 | 11 |
| 29733 | M13 240634 | 11.1 | South American/UK strain | UK | 2013 | W | 5 | 2 | 11 |
| 29734 | M13 240538 | 11.1 | South American/UK strain | UK | 2013 | W | 5 | 2 | 11 |
| 29735 | M13 240515 | 11.1 | South American/UK strain | UK | 2013 | W | 5 | 2 | 11 |
| 29736 | M13 240600 | 11.1 | South American/UK strain | UK | 2013 | W | 5 | 2 | 11 |
| 29737 | M13 240553 | 11.1 | South American/UK strain | UK | 2013 | W | 5 | 2 | 11 |
| 30136 | M13 240635 | 11.1 | South American/UK strain | UK | 2013 | W | 5 | 2 | 11 |
| 30137 | M13 240637 | 11.1 | South American/UK strain | UK | 2013 | W | 5 | 2 | 11 |
| 30138 | M13 240649 | 11.1 | South American/UK strain | UK | 2013 | W | 5 | 2 | 11 |
| 30139 | M13 240655 | 11.1 | South American/UK strain | UK | 2013 | W | 5 | 2 | 11 |
| 30140 | M13 240657 | 11.1 | South American/UK strain | UK | 2013 | W | 5 | 2 | 11 |
| 30141 | M13 240664 | 11.1 | South American/UK strain | UK | 2013 | W | 5 | 2 | 11 |
| 30142 | M13 240666 | 11.1 | South American/UK strain | UK | 2013 | W | 5 | 2 | 11 |
| 30143 | M13 240667 | 11.1 | South American/UK strain | UK | 2013 | W | 5 | 2 | 11 |
| 30144 | M13 240668 | 11.1 | South American/UK strain | UK | 2013 | W | 5 | 2 | 11 |
| 30145 | M13 240672 | 11.1 | South American/UK strain | UK | 2013 | W | 5 | 2 | 11 |
| 30146 | M13 240678 | 11.1 | South American/UK strain | UK | 2013 | W | 5-1 | 10-4 | 11 |
| 30147 | M13 240680 | 11.1 | South American/UK strain | UK | 2013 | W | 5 | 2 | 11 |
| 30148 | M13 240681 | 11.1 | South American/UK strain | UK | 2013 | W | 5 | 2 | 11 |
| 30150 | M13 240724 | 11.1 | South American/UK strain | UK | 2013 | W | 5 | 2 | 11 |
| 30151 | M13 240726 | 11.1 | South American/UK strain | UK | 2013 | W | 5 | 2 | 11 |
| 30152 | M13 240730 | 11.1 | South American/UK strain | UK | 2013 | W | 5 | 2 | 11 |
| 30153 | M13 240732 | 11.1 | South American/UK strain | UK | 2013 | W | 5 | 2 | 11 |
| 30154 | M14 240001 | 11.1 | South American/UK strain | UK | 2014 | W | 5 | 2 | 11 |
| 30155 | M14 240002 | 11.1 | South American/UK strain | UK | 2014 | W | 5 | 2 | 11 |
| 30156 | M14 240007 | 11.1 | South American/UK strain | UK | 2014 | W | 5 | 2 | 11 |
| 30157 | M14 240013 | 11.1 | South American/UK strain | UK | 2014 | W | 5 | 2 | 11 |
| 30158 | M14 240019 | 11.1 | South American/UK strain | UK | 2014 | W | 5 | 2 | 11 |
| 30159 | M14 240022 | 11.1 | South American/UK strain | UK | 2014 | W | 5 | 2 | 8621 |
| 30160 | M14 240026 | 11.1 | South American/UK strain | UK | 2014 | W | 5 | 2 | 11 |
| 30161 | M14 240029 | 11.1 | South American/UK strain | UK | 2014 | W | 5 | 2 | 11 |
| 30162 | M14 240031 | 11.1 | South American/UK strain | UK | 2014 | W | 5 | 2 | 11 |
| 30163 | M14 240042 | 11.1 | South American/UK strain | UK | 2014 | W | 5 | 2 | 11 |
| 30164 | M14 240043 | 11.1 | South American/UK strain | UK | 2014 | W | 5 | 2 | 11 |
| 30165 | M14 240052 | 11.1 | South American/UK strain | UK | 2014 | W | 5 | 2 | 11 |
| 30166 | M14 240053 | 11.1 | South American/UK strain | UK | 2014 | W | 5 | 2 | 10651 |
| 30167 | M14 240054 | 11.1 | South American/UK strain | UK | 2014 | W | 5 | 2 | 11 |
| 30168 | M14 240072 | 11.1 | South American/UK strain | UK | 2014 | W | 5 | 2 | 11 |
| 30169 | M14 240082 | 11.1 | South American/UK strain | UK | 2014 | W | 5 | 2 | 11 |
| 30170 | M14 240086 | 11.1 | South American/UK strain | UK | 2014 | W | 5 | 2 | 11 |
| 31169 | M14 240448 | 11.1 | South American/UK strain | France uk tourist | 2014 | W | 5 | 2 | 11 |
| 31166 | M14 240445 | 11.1 | South American/UK strain | France recent | 2014 | W | 5 | 2 | 11 |
| 26918 | 10007v1 | 11.1 | South American/UK strain (carriage study) | UK | 2010 | W | 5 | 2 | 11 |
| 26919 | 10117v2 | 11.1 | South American/UK strain (carriage study) | UK | 2010 | W | 5 | 2 | 11 |
| 26925 | 10507v4 | 11.1 | South American/UK strain (carriage study) | UK | 2011 | W | 5 | 2 | 11 |
| 26930 | 10569v5 | 11.1 | South American/UK strain (carriage study) | UK | 2011 | W | 5 | 2 | 11 |
| 26931 | 40118v3 | 11.1 | South American/UK strain (carriage study) | UK | 2011 | W | 5 | NV | 11 |
| 26933 | 40161v1 | 11.1 | South American/UK strain (carriage study) | UK | 2010 | W | 5 | 2 | 11 |
| 26944 | 70045v4b | 11.1 | South American/UK strain (carriage study) | UK | 2011 | W | 5 | 2 | 11 |
| 26945 | 70306v6 | 11.1 | South American/UK strain (carriage study) | UK | 2011 | W | 5 | 2 | 11 |
| 26946 | 70322v3 | 11.1 | South American/UK strain (carriage study) | UK | 2011 | W | 5 | 2 | 11 |
| 26949 | 80046v4b | 11.1 | South American/UK strain (carriage study) | UK | 2011 | W | 5 | 2 | 10284 |
| 26952 | 80164v1 | 11.1 | South American/UK strain (carriage study) | UK | 2010 | W | 5 | 2 | 11 |
| 26957 | 90021v3 | 11.1 | South American/UK strain (carriage study) | UK | 2010 | W | 5 | 2 | 11 |
| 26959 | 90022v2 | 11.1 | South American/UK strain (carriage study) | UK | 2010 | W | 5 | 2 | 11 |
| 26962 | 90028v1 | 11.1 | South American/UK strain (carriage study) | UK | 2010 | W | 5 | 2 | 11 |
| 26963 | 90034v4 | 11.1 | South American/UK strain (carriage study) | UK | 2011 | W | 5 | 2 | 11 |
| 26965 | 90058v2 | 11.1 | South American/UK strain (carriage study) | UK | 2010 | W | 5 | 2 | 11 |
| 26968 | 90066v1 | 11.1 | South American/UK strain (carriage study) | UK | 2010 | W | 5 | 2 | 11 |
| 26970 | 90092v1 | 11.1 | South American/UK strain (carriage study) | UK | 2010 | W | 5 | 2 | 11 |
| 26974 | 90102v3 | 11.1 | South American/UK strain (carriage study) | UK | 2011 | W | 5 | 2 | 11 |
| 26978 | 90111v4 | 11.1 | South American/UK strain (carriage study) | UK | 2011 | W | 5 | 2 | 11 |
| 26980 | 90119v2 | 11.1 | South American/UK strain (carriage study) | UK | 2010 | W | 5 | 2 | 11 |
| 26984 | 90124v4 | 11.1 | South American/UK strain (carriage study) | UK | 2011 | W | 5 | 2 | 11 |
| 26989 | 90136v3 | 11.1 | South American/UK strain (carriage study) | UK | 2010 | W | 5 | 2 | 11 |
| 26991 | 90130v3 | 11.1 | South American/UK strain (carriage study) | UK | 2010 | W | 5 | 2 | 11 |
| 26992 | 90142v1 | 11.1 | South American/UK strain (carriage study) | UK | 2010 | W | 5 | 2 | 11 |
| 26993 | 90149v1 | 11.1 | South American/UK strain (carriage study) | UK | 2010 | W | 7-2 | 14 | 11 |
| 26994 | 90174v1 | 11.1 | South American/UK strain (carriage study) | UK | 2010 | W | 5 | 2 | 11 |
| 26995 | 90182v1 | 11.1 | South American/UK strain (carriage study) | UK | 2010 | W | 5 | 2 | 11 |
| 26997 | 90075v2 | 11.1 | South American/UK strain (carriage study) | UK | 2010 | W | 5 | 2 | 11 |
|  |  |  |  |  |  |  |  |  |  |
| 31148 | M14 240411 | 11.1 | South American/UK strain | Argentina | 2008 | W | 5 | 2 | 11 |
| 31149 | M14 240412 | 11.1 | South American/UK strain | Argentina | 2010 | W | 5 | 2 | 11 |
| 31150 | M14 240413 | 11.1 | South American/UK strain | Argentina | 2011 | W | 5 | 2 | 10856 |
| 31151 | M14 240414 | 11.1 | South American/UK strain | Argentina | 2011 | W | 5 | 2 | 10857 |
| 31152 | M14 240415 | 11.1 | South American/UK strain | Argentina | 2012 | W | 5 | 2 | 11 |
| 20057 | M10 240671 | 11.1 | South American/UK strain | UK | 2010 | W | 5 | 2 | 11 |
| 31168 | M14 240447 | 11.1 | South American/UK strain | France uk tourist | 2014 | W | 5 | 2 | 11 |
|  |  |  |  |  |  |  |  |  |  |
| 31155 | M14 240416 | 11.1 | South American/UK strain | Brazil | 2011 | W | 5 | 2 | 11 |
| 31156 | M14 240417 | 11.1 | South American/UK strain | Brazil | 2011 | W | 5 | 2 | 11 |
| 31157 | M14 240418 | 11.1 | South American/UK strain | Brazil | 2011 | W | 5 | 2 | 11 |
| 31159 | M14 240420 | 11.1 | South American/UK strain | Brazil | 2008 | W | 5 | 2 | 11 |
| 31160 | M14 240421 | 11.1 | South American/UK strain | Brazil | 2009 | W | 5 | 2 | * |
| 31161 | M14 240422 | 11.1 | South American/UK strain | Brazil | 2008 | W | 5 | 2 | 11 |
| 31158 | M14 240419 | 11.1 | South American/UK strain | Brazil | 2010 | W | 5 | 2 | 11 |
|  |  |  |  |  |  |  |  |  |  |
| 29324 | 11361 | 11.1 | lineage 11.1 intermediate region | South Africa | 2003 | W | 5 | 2 | 11 |
| 29325 | 11665 | 11.1 | lineage 11.1 intermediate region | South Africa | 2003 | W | 5 | 2 | 11 |
| 29358 | 15512 | 11.1 | lineage 11.1 intermediate region | South Africa | 2004 | W | 5 | 2 | 11 |
| 29331 | 4846 | 11.1 | lineage 11.1 intermediate region | South Africa | 2005 | W | 5 | 2 | 11 |
| 29362 | 10114 | 11.1 | lineage 11.1 intermediate region | South Africa | 2006 | W | 5 | 2 | 11 |
| 29426 | 7720 | 11.1 | lineage 11.1 intermediate region | South Africa | 2006 | W | 5 | 2 | 11 |
| 29409 | 12782 | 11.1 | lineage 11.1 intermediate region | South Africa | 2007 | W | 5 | 2 | 11 |
| 29376 | 24581 | 11.1 | lineage 11.1 intermediate region | South Africa | 2009 | W | 5 | 2 | 11 |
| 29399 | 24344 | 11.1 | lineage 11.1 intermediate region | South Africa | 2009 | W | 5 | 2 | 11 |
| 29328 | 30301 | 11.1 | lineage 11.1 intermediate region | South Africa | 2010 | W | 5 | 2 | 11 |
| 29349 | 28444 | 11.1 | lineage 11.1 intermediate region | South Africa | 2010 | W | 5 | 2 | 11 |
| 29364 | 33509 | 11.1 | lineage 11.1 intermediate region | South Africa | 2011 | W | 5 | 2 | 11 |
| 29407 | 33822 | 11.1 | lineage 11.1 intermediate region | South Africa | 2011 | W | 5 | 2 | 11 |
| 29372 | 38108 | 11.1 | lineage 11.1 intermediate region | South Africa | 2012 | W | 5 | 2 | 11 |
| 29423 | 41043 | 11.1 | lineage 11.1 intermediate region | South Africa | 2013 | W | 5 | 2 | 11 |
|  |  |  |  |  |  |  |  |  |  |
| 29334 | 11009 | 11.1 | lineage 11.1 intermediate region | South Africa | 2003 | W | 5-1 | 2 | 11 |
| 29341 | 10012 | 11.1 | lineage 11.1 intermediate region | South Africa | 2003 | W | 5-1 | 2 | 11 |
| 29366 | 10992 | 11.1 | lineage 11.1 intermediate region | South Africa | 2003 | W | 5-1 | 2 | 11 |
| 29394 | 14673 | 11.1 | lineage 11.1 intermediate region | South Africa | 2004 | W | 5-1 | 2 | 11 |
| 21581 | SA_serogroup W_NM9 | 11.1 | lineage 11.1 intermediate region | South Africa | 2007 | W | 5-1 | 2 | 11 |
| 29340 | 17548 | 11.1 | lineage 11.1 intermediate region | South Africa | 2008 | W | 5-1 | 2 | 11 |
| 29441 | 19733 | 11.1 | lineage 11.1 intermediate region | South Africa | 2008 | W | 5-1 | 2 | 11 |
| 29385 | 21296 | 11.1 | lineage 11.1 intermediate region | South Africa | 2009 | W | 5-1 | 2 | 11 |
| 29420 | 31810 | 11.1 | lineage 11.1 intermediate region | South Africa | 2011 | W | 5-1 | 2 | 11 |
|  |  |  |  |  |  |  |  |  |  |
| 29648 | M98 252422 | 11.1 | lineage 11.1 intermediate region | UK | 1975 | W | 5 | 2 | 11 |
| 29649 | M98 252412 | 11.1 | lineage 11.1 intermediate region | UK | 1975 | W | 5 | 2 | 11 |
| 29651 | M98 252407 | 11.1 | lineage 11.1 intermediate region | UK | 1975 | W | 5 | 2 | 11 |
| 29652 | M98 252491 | 11.1 | lineage 11.1 intermediate region | UK | 1975 | W | 5 | 2 | 473 |
| 29653 | M98 252431 | 11.1 | lineage 11.1 intermediate region | UK | 1975 | W | 5 | 2 | 11 |
| 29650 | M98 252490 | 11.1 | lineage 11.1 intermediate region | UK | 1975 | W | 5 | 2 | 473 |
|  |  |  |  |  |  |  |  |  |  |
| 30234 | M96 252316 | 11.1 | lineage 11.1 intermediate region | UK | 1996 | W | 5 | 2 | 11 |
| 30237 | M96 255688 | 11.1 | lineage 11.1 intermediate region | UK | 1996 | W | 5 | 2 | 11 |
| 30251 | H87 000098 | 11.1 | lineage 11.1 intermediate region | UK | 1987 | W | 5 | 2 | 11 |
| 30253 | K89 000575 | 11.1 | lineage 11.1 intermediate region | UK | 1989 | W | 5 | 2 | 11 |
| 30255 | L90 002191 | 11.1 | lineage 11.1 intermediate region | UK | 1990 | W | 19 | 15 | 11 |
| 30178 | M98 250031 | 11.1 | lineage 11.1 intermediate region | UK | 1998 | W | 5 | 2 | 1237 |
| 29709 | M07 240774 | 11.1 | lineage 11.1 intermediate region | UK | 2007 | W | 5 | 2 | 247 |
| 29710 | M07 240922 | 11.1 | lineage 11.1 intermediate region | UK | 2007 | W | 5-2 | 10 | 247 |
|  |  |  |  |  |  |  |  |  |  |
| 29273 | BM49 | 11.1 | lineage 11.1 proximal region | Greece | 1996 | B | 5 | 2 | 11 |
| 436 | NG P20 | 11.1 | lineage 11.1 proximal region | Norway | 1969 | B | 5 | 2 | 11 |
| 349 | 38VI | 11.1 | lineage 11.1 proximal region | USA | 1964 | B | 5 | 2 | 11 |
| 344 | F1576 | 11.1 | lineage 11.1 proximal region | Ghana | 1984 | C | 5 | 2 | 11 |
| 343 | 500 | 11.1 | lineage 11.1 proximal region | Italy | 1984 | C | 5 | 2 | 11 |
| 30263 | M02 242035 | 11.1 | lineage 11.1 proximal region | UK | 1970 | C | 5 | 2 | 2735 |
| 30259 | M02 242030 | 11.1 | lineage 11.1 proximal region | UK | 1970 | C | 5 | 2 | 11 |
| 30264 | M02 242062 | 11.1 | lineage 11.1 proximal region | UK | 1970 | C | 5 | 2 | 11 |
| 30244 | F85 0000704 | 11.1 | lineage 11.1 proximal region | UK | 1985 | C | 5 | 2 | 11 |
| 314 | D1 | 11.1 | lineage 11.1 proximal region | Mali | 1989 | C | 5 | 2-1 | 11 |
| 468 | BRAZ10 | 11.1 | lineage 11.1 proximal region | Brazil | 1976 | C | 5-1 | 10-1 | 11 |
|  |  |  |  |  |  |  |  |  |  |
| 21335 | M12 240069 | 11.1 | lineage 11.1 proximal region | UK | 2012 | B | 5 | 2 | 11 |
| 29643 | M97 253597 | 11.1 | lineage 11.1 proximal region | UK | 1997 | C | 5 | 2 | 67 |
| 665 | 2845 | 11.1 | lineage 11.1 proximal region | UK | 1997 | C | 5 | 2 | 67 |
| 29760 | M98 252222 | 11.1 | lineage 11.1 proximal region | UK | 1998 | C | 5 | 2 | 67 |
| 29761 | M98 252223 | 11.1 | lineage 11.1 proximal region | UK | 1998 | C | 5 | 2 | 67 |
| 29762 | M98 252224 | 11.1 | lineage 11.1 proximal region | UK | 1998 | C | 5 | 2 | 67 |
| 29578 | M04 240129 | 11.1 | lineage 11.1 proximal region | UK | 2004 | C | 5 | 2 | 11 |
| 21196 | M11 240785 | 11.1 | lineage 11.1 proximal region | UK | 2011 | C | 5 | 2 | 11 |
|  |  |  |  |  |  |  |  |  |  |
| 30287 | M14 240184 | 11.1 | lineage 11.1 proximal region | Canada | 2009 | C | 5 | 2 | 11 |
| 26859 | 12029_2011 | 11.1 | lineage 11.1 proximal region | Ireland | 2011 | C | 5 | 2 | 11 |
| 29896 | M13 240382 | 11.1 | lineage 11.1 proximal region | Malta | 2013 | C | 5 | 2 | 11 |
| 29901 | M98 250863 | 11.1 | lineage 11.1 proximal region | UK | 1998 | C | 5 | 2 | 11 |
| 29646 | M98 253000 | 11.1 | lineage 11.1 proximal region | UK | 1998 | C | 5 | 2 | 11 |
| 29611 | M06 240019 | 11.1 | lineage 11.1 proximal region | UK | 2006 | C | 5 | 2 | 11 |
| 29626 | M07 240686 | 11.1 | lineage 11.1 proximal region | UK | 2007 | C | 5 | 2 | 7779 |
| 29638 | M08 240026 | 11.1 | lineage 11.1 proximal region | UK | 2008 | C | 5 | 2 | 11 |
| 29891 | M10 240229 | 11.1 | lineage 11.1 proximal region | UK | 2010 | C | 5 | 2 | 11 |
| 20234 | M11 240076 | 11.1 | lineage 11.1 proximal region | UK | 2011 | C | 5 | 2 | 11 |
| 21458 | M12 240255 | 11.1 | lineage 11.1 proximal region | UK | 2012 | C | 5 | 2 | 11 |
| 28080 | M12 240639 | 11.1 | lineage 11.1 proximal region | UK | 2012 | C | 5 | 2 | 11 |
| 28084 | M12 240716 | 11.1 | lineage 11.1 proximal region | UK | 2012 | C | 5 | 2 | 11 |
| 28089 | M13 240008 | 11.1 | lineage 11.1 proximal region | UK | 2013 | C | 5 | 2 | 11 |
| 28092 | M13 240068 | 11.1 | lineage 11.1 proximal region | UK | 2013 | C | 5 | 2 | 11 |
| 28102 | M13 240401 | 11.1 | lineage 11.1 proximal region | UK | 2013 | C | 5 | 2 | 11 |
| 28106 | M13 240460 | 11.1 | lineage 11.1 proximal region | UK | 2013 | C | 5 | 2 | 11 |
| 29895 | M13 240496 | 11.1 | lineage 11.1 proximal region | UK | 2013 | C | 5 | 2 | 11 |
| 29898 | M13 240514 | 11.1 | lineage 11.1 proximal region | UK | 2013 | C | 5 | 2 | 11 |
| 30214 | M14 240100 | 11.1 | lineage 11.1 proximal region | UK | 2014 | C | 5 | 2 | 11 |
| 30215 | M14 240107 | 11.1 | lineage 11.1 proximal region | UK | 2014 | C | 5 | 2 | 11 |
| 30286 | M14 240183 | 11.1 | lineage 11.1 proximal region | Canada | 2008 | C | 5 | 2 | 11 |
|  |  |  |  |  |  |  |  |  |  |
| 369 | M597 | 11.1 | lineage 11.1 proximal region | Israel | 1988 | C | 5 | 2-1 | 11 |
| 29616 | M06 240340 | 11.1 | lineage 11.1 proximal region | Malta | 2006 | C | 5 | 2-1 | 11 |
| 507 | MA-5756 | 11.1 | lineage 11.1 proximal region | Spain | 1985 | C | 5 | 2-1 | 11 |
| 30239 | F85 0000001 | 11.1 | lineage 11.1 proximal region | UK | 1985 | C | 5 | 2-1 | 11 |
| 30240 | F85 0000045 | 11.1 | lineage 11.1 proximal region | UK | 1985 | C | 5 | 2-1 | 11 |
| 30241 | F85 0000076 | 11.1 | lineage 11.1 proximal region | UK | 1985 | C | 5 | 2-1 | 11 |
| 30243 | F85 0000671 | 11.1 | lineage 11.1 proximal region | UK | 1985 | C | 5 | 2-1 | 11 |
| 30245 | F85 0000746 | 11.1 | lineage 11.1 proximal region | UK | 1985 | C | 5 | 2-1 | 11 |
| 30242 | F85 0000171 | 11.1 | lineage 11.1 proximal region | UK | 1985 | C | 5 | 2-1 | 11 |
| 391 | 90/18311 | 11.1 | lineage 11.1 proximal region | UK | 1990 | C | 5 | 2-1 | 11 |
| 29596 | M05 240080 | 11.1 | lineage 11.1 proximal region | UK | 2005 | C | 5 | 2-1 | 11 |
| 29907 | M98 252898 | 11.1 | lineage 11.1 proximal region | UK | 1998 | C | 5-1 | 10-4 | 11 |
| 667 | 2842 | 11.1 | lineage 11.1 proximal region | UK | 1997 | C | 19-3 | 15 | 51 |
|  |  |  |  |  |  |  |  |  |  |
| 30276 | M01 240364 | 11.1 | lineage 11.1 proximal region | UK | 2001 | B | 5 | 2 | 11 |
| 29849 | M01 240493 | 11.1 | lineage 11.1 proximal region | Ireland | 2001 | C | 5 | 2 | 11 |
| 29858 | M01 242751 | 11.1 | lineage 11.1 proximal region | Ireland | 2001 | C | 5 | 2 | 11 |
| 29863 | M02 240385 | 11.1 | lineage 11.1 proximal region | Ireland | 2002 | C | 5 | 2 | 11 |
| 29877 | M03 241704 | 11.1 | lineage 11.1 proximal region | Ireland | 2003 | C | 5 | 2 | 11 |
| 30192 | M96 255871 | 11.1 | lineage 11.1 proximal region | UK | 1996 | C | 5 | 2 | 11 |
| 30193 | M96 255976 | 11.1 | lineage 11.1 proximal region | UK | 1996 | C | 5 | 2 | 11 |
| 29840 | M00 241761 | 11.1 | lineage 11.1 proximal region | UK | 2000 | C | 5 | 2 | 11 |
| 29665 | M00 241315 | 11.1 | lineage 11.1 proximal region | UK | 2000 | C | 5 | 2 | 11 |
| 29865 | M02 240206 | 11.1 | lineage 11.1 proximal region | UK | 2002 | C | 5 | 2 | 11 |
| 29693 | M02 241029 | 11.1 | lineage 11.1 proximal region | UK | 2002 | C | 5 | 2 | 11 |
| 29978 | M03 241465 | 11.1 | lineage 11.1 proximal region | UK | 2003 | C | 5 | 2 | 11 |
| 698 | FAM18 | 11.1 | lineage 11.1 proximal region | USA | 1983 | C | 5 | 2 | 11 |
| 669 | 2846 | 11.1 | lineage 11.1 proximal region | UK | 1997 | C | deleted | deleted | 52 |
| 29610 | M05 241361 | 11.1 | lineage 11.1 proximal region | UK | 2005 | C | deleted | deleted | 11 |
| 29612 | M06 240067 | 11.1 | lineage 11.1 proximal region | UK | 2006 | C | deleted | deleted | 11 |
|  |  |  |  |  |  |  |  |  |  |
| 29390 | 37450 | 11.2 | n/a | South Africa | 2012 | C | 5-1 | 10-6 | 11 |
| 29670 | M00 243009 | 11.2 | n/a | UK | 2000 | B | 5-1 | 10-8 | 11 |
| 30051 | M03 241710 | 11.2 | n/a | UK | 2003 | B | 5-1 | 10-8 | 11 |
| 21311 | M12 240033 | 11.2 | n/a | UK | 2012 | B | 5-1 | 10-8 | 11 |
| 21330 | M12 240061 | 11.2 | n/a | UK | 2012 | B | 5-1 | 10-8 | 11 |
| 27803 | M12 240682 | 11.2 | n/a | UK | 2012 | B | 5-1 | 10-8 | 11 |
| 27992 | M13 240199 | 11.2 | n/a | UK | 2013 | B | 5-1 | 10-8 | 11 |
| 26824 | 12025_2010 | 11.2 | n/a | Ireland | 2010 | C | 5-1 | 10-8 | 11 |
| 29600 | M05 240316 | 11.2 | n/a | UK | 2005 | C | 5-1 | 10-8 | 11 |
| 29608 | M05 241164 | 11.2 | n/a | UK | 2005 | C | 5-1 | 10-8 | 11 |
| 29613 | M06 240167 | 11.2 | n/a | UK | 2006 | C | 5-1 | 10-8 | 11 |
| 29620 | M06 240537 | 11.2 | n/a | UK | 2006 | C | 5-1 | 10-8 | 11 |
| 29625 | M07 240006 | 11.2 | n/a | UK | 2007 | C | 5-1 | 10-8 | 11 |
| 29888 | M09 240249 | 11.2 | n/a | UK | 2009 | C | 5-1 | 10-8 | 11 |
| 21134 | M11 240502 | 11.2 | n/a | UK | 2011 | C | 5-1 | 10-8 | 11 |
| 28090 | M13 240022 | 11.2 | n/a | UK | 2013 | C | 5-1 | 10-8 | 11 |
|  |  |  |  |  |  |  |  |  |  |
| 29990 | M00 240815 | 11.2 | n/a | UK | 2000 | B | 5-1 | 10-8 | 11 |
| 30003 | M01 240663 | 11.2 | n/a | UK | 2001 | B | 5-1 | 10-8 | 11 |
| 30000 | M01 240622 | 11.2 | n/a | UK | 2001 | B | 5-1 | 10-8 | 11 |
| 30048 | M02 241095 | 11.2 | n/a | UK | 2002 | B | 5-1 | 10-8 | 11 |
| 30046 | M02 240038 | 11.2 | n/a | UK | 2002 | B | 5-1 | 10-8 | 1789 |
| 30058 | M08 240227 | 11.2 | n/a | UK | 2008 | B | 5-1 | 10-8 | 11 |
| 30059 | M08 240819 | 11.2 | n/a | UK | 2008 | B | 5-1 | 10-8 | 11 |
| 20066 | M10 240684 | 11.2 | n/a | UK | 2010 | B | 5-1 | 10-8 | 11 |
| 20215 | M11 240056 | 11.2 | n/a | UK | 2011 | B | 5-1 | 10-8 | 11 |
| 21237 | M11 241016 | 11.2 | n/a | UK | 2011 | B | 5-1 | 10-8 | 11 |
| 30288 | M14 240185 | 11.2 | n/a | Canada | 2001 | C | 5-1 | 10-8 | 11 |
| 29607 | M05 240990 | 11.2 | n/a | Ireland | 2005 | C | 5-1 | 10-8 | 11 |
| 29618 | M06 240493 | 11.2 | n/a | Ireland | 2006 | C | 5-1 | 10-8 | 11 |
| 19365 | K1207 | 11.2 | n/a | Italy | 2007 | C | 5-1 | 10-8 | 11 |
| 19505 | S0108 | 11.2 | n/a | Italy | 2008 | C | 5-1 | 10-8 | 11 |
| 30130 | M05 240857 | 11.2 | n/a | Spain | 1999 | C | 5-1 | 10-8 | 11 |
| 30131 | M05 240858 | 11.2 | n/a | Spain | 2001 | C | 5-1 | 10-8 | 11 |
| 30126 | M05 240853 | 11.2 | n/a | Spain | 2001 | C | 5-1 | 10-8 | 11 |
| 30132 | M05 240859 | 11.2 | n/a | Spain | 2001 | C | 5-1 | 10-8 | 11 |
| 30134 | M05 240861 | 11.2 | n/a | Spain | 2002 | C | 5-1 | 10-8 | 11 |
| 30188 | M96 255774 | 11.2 | n/a | UK | 1996 | C | 5-1 | 10-8 | 11 |
| 30185 | M96 255770 | 11.2 | n/a | UK | 1996 | C | 5-1 | 10-8 | 11 |
| 29644 | M98 250473 | 11.2 | n/a | UK | 1998 | C | 5-1 | 10-8 | 11 |
| 29910 | M98 253743 | 11.2 | n/a | UK | 1998 | C | 5-1 | 10-8 | 11 |
| 29750 | M98 250786 | 11.2 | n/a | UK | 1998 | C | 5-1 | 10-8 | 11 |
| 29751 | M98 250787 | 11.2 | n/a | UK | 1998 | C | 5-1 | 10-8 | 11 |
| 29752 | M98 250788 | 11.2 | n/a | UK | 1998 | C | 5-1 | 10-8 | 11 |
| 29658 | M99 241667 | 11.2 | n/a | UK | 1999 | C | 5-1 | 10-8 | 11 |
| 30206 | M99 240772 | 11.2 | n/a | UK | 1999 | C | 5-1 | 10-8 | 11 |
| 29654 | M99 240321 | 11.2 | n/a | UK | 1999 | C | 5-1 | 10-8 | 11 |
| 29663 | M00 240609 | 11.2 | n/a | UK | 2000 | C | 5-1 | 10-8 | 11 |
| 29667 | M00 241657 | 11.2 | n/a | UK | 2000 | C | 5-1 | 10-8 | 11 |
| 29668 | M00 241980 | 11.2 | n/a | UK | 2000 | C | 5-1 | 10-8 | 11 |
| 29664 | M00 240972 | 11.2 | n/a | UK | 2000 | C | 5-1 | 10-8 | 11 |
| 29851 | M01 240850 | 11.2 | n/a | UK | 2001 | C | 5-1 | 10-8 | 11 |
| 29847 | M01 240526 | 11.2 | n/a | UK | 2001 | C | 5-1 | 10-8 | 3463 |
| 29977 | M03 241273 | 11.2 | n/a | UK | 2003 | C | 5-1 | 10-8 | 11 |
| 29976 | M03 241118 | 11.2 | n/a | UK | 2003 | C | 5-1 | 10-8 | 1055 |
| 29574 | M04 240020 | 11.2 | n/a | UK | 2004 | C | 5-1 | 10-8 | 11 |
| 29582 | M04 240323 | 11.2 | n/a | UK | 2004 | C | 5-1 | 10-8 | 11 |
| 29580 | M04 240235 | 11.2 | n/a | UK | 2004 | C | 5-1 | 10-8 | 11 |
| 29581 | M04 240259 | 11.2 | n/a | UK | 2004 | C | 5-1 | 10-8 | 11 |
| 29572 | M04 240003 | 11.2 | n/a | UK | 2004 | C | 5-1 | 10-8 | 11 |
| 29587 | M04 240931 | 11.2 | n/a | UK | 2004 | C | 5-1 | 10-8 | 11 |
| 29595 | M04 241722 | 11.2 | n/a | UK | 2004 | C | 5-1 | 10-8 | 11 |
| 29586 | M04 240752 | 11.2 | n/a | UK | 2004 | C | 5-1 | 10-8 | 11 |
| 29583 | M04 240335 | 11.2 | n/a | UK | 2004 | C | 5-1 | 10-8 | 11 |
| 29585 | M04 240594 | 11.2 | n/a | UK | 2004 | C | 5-1 | 10-8 | 11 |
| 29584 | M04 240348 | 11.2 | n/a | UK | 2004 | C | 5-1 | 10-8 | 11 |
| 29601 | M05 240490 | 11.2 | n/a | UK | 2005 | C | 5-1 | 10-8 | 11 |
| 29619 | M06 240536 | 11.2 | n/a | UK | 2006 | C | 5-1 | 10-8 | 11 |
| 29624 | M06 240168 | 11.2 | n/a | UK | 2006 | C | 5-1 | 10-8 | 11 |
| 29615 | M06 240183 | 11.2 | n/a | UK | 2006 | C | 5-1 | 10-8 | 11 |
| 29629 | M07 240954 | 11.2 | n/a | UK | 2007 | C | 5-1 | 10-8 | 5149 |
| 29633 | M07 240210 | 11.2 | n/a | UK | 2007 | C | 5-1 | 10-8 | 5149 |
| 29628 | M07 240924 | 11.2 | n/a | UK | 2007 | C | 5-1 | 10-8 | 491 |
| 29636 | M07 240541 | 11.2 | n/a | UK | 2007 | C | 5-1 | 10-8 | 11 |
| 29637 | M07 240593 | 11.2 | n/a | UK | 2007 | C | 5-1 | 10-8 | 11 |
| 29631 | M07 241093 | 11.2 | n/a | UK | 2007 | C | 5-1 | 10-8 | 11 |
| 29640 | M08 240201 | 11.2 | n/a | UK | 2008 | C | 5-1 | 10-8 | 11 |
| 27089 | LNP24198 | 11.2 | n/a | France | 2007 | C | 7-1 | 1 | 11 |
|  |  |  |  |  |  |  |  |  |  |
| 26822 | LNP26948 | 11.2 | n/a | France | 2012 | C | 5-1 | 10-1 | 10482 |
| 29639 | M08 240185 | 11.2 | n/a | UK | 2008 | C | 5-1 | 10-1 | 11 |
| 21110 | M11 240469 | 11.2 | n/a | UK | 2011 | B | 5-1 | 10-8 | 10260 |
| 26733 | LNP27256 | 11.2 | n/a | France | 2013 | C | 5-1 | 10-8 | 11 |
| 26821 | LNP27257 | 11.2 | n/a | France | 2013 | C | 5-1 | 10-8 | 11 |
| 29889 | M09 240241 | 11.2 | n/a | Malta | 2009 | C | 5-1 | 10-8 | 52 |
| 21185 | M11 240768 | 11.2 | n/a | Malta | 2011 | C | 5-1 | 10-8 | 11 |
| 28800 | LNP27366 | 11.2 | n/a | Poland | 2012 | C | 5-1 | 10-8 | 11 |
| 29621 | M06 240625 | 11.2 | n/a | UK | 2006 | C | 5-1 | 10-8 | 11 |
| 29635 | M07 240351 | 11.2 | n/a | UK | 2007 | C | 5-1 | 10-8 | 11 |
| 29627 | M07 240723 | 11.2 | n/a | UK | 2007 | C | 5-1 | 10-8 | 11 |
| 20155 | M10 240818 | 11.2 | n/a | UK | 2010 | C | 5-1 | 10-8 | 11 |
| 29890 | M10 240001 | 11.2 | n/a | UK | 2010 | C | 5-1 | 10-8 | 11 |
| 21232 | M11 240994 | 11.2 | n/a | UK | 2011 | C | 5-1 | 10-8 | 11 |
| 20329 | M11 240247 | 11.2 | n/a | UK | 2011 | C | 5-1 | 10-8 | 11 |
| 20359 | M11 240294 | 11.2 | n/a | UK | 2011 | C | 5-1 | 10-8 | 11 |
| 21253 | M11 241039 | 11.2 | n/a | UK | 2011 | C | 5-1 | 10-8 | 11 |
| 21208 | M11 240941 | 11.2 | n/a | UK | 2011 | C | 5-1 | 10-8 | 11 |
| 21256 | M11 241043 | 11.2 | n/a | UK | 2011 | C | 5-1 | 10-8 | 11 |
| 21407 | M12 240177 | 11.2 | n/a | UK | 2012 | C | 5-1 | 10-8 | 11 |
| 21359 | M12 240101 | 11.2 | n/a | UK | 2012 | C | 5-1 | 10-8 | 11 |
| 21364 | M12 240111 | 11.2 | n/a | UK | 2012 | C | 5-1 | 10-8 | 11 |
| 28085 | M12 240776 | 11.2 | n/a | UK | 2012 | C | 5-1 | 10-8 | 11 |
| 28083 | M12 240698 | 11.2 | n/a | UK | 2012 | C | 5-1 | 10-8 | 11 |
| 21419 | M12 240202 | 11.2 | n/a | UK | 2012 | C | 5-1 | 10-8 | 11 |
| 28082 | M12 240679 | 11.2 | n/a | UK | 2012 | C | 5-1 | 10-8 | 11 |
| 28103 | M13 240402 | 11.2 | n/a | UK | 2013 | C | 5-1 | 10-8 | 11 |
| 28099 | M13 240282 | 11.2 | n/a | UK | 2013 | C | 5-1 | 10-8 | 11 |
| 28105 | M13 240440 | 11.2 | n/a | UK | 2013 | C | 5-1 | 10-8 | 11 |
| 30210 | M13 240691 | 11.2 | n/a | UK | 2013 | C | 5-1 | 10-8 | 11 |
| 28094 | M13 240162 | 11.2 | n/a | UK | 2013 | C | 5-1 | 10-8 | 11 |
| 28093 | M13 240155 | 11.2 | n/a | UK | 2013 | C | 5-1 | 10-8 | 11 |
| 28100 | M13 240295 | 11.2 | n/a | UK | 2013 | C | 5-1 | 10-8 | 11 |
| 28097 | M13 240254 | 11.2 | n/a | UK | 2013 | C | 5-1 | 10-8 | 11 |
| 26732 | M13 240559 | 11.2 | n/a | UK | 2013 | C | 5-1 | 10-8 | 11 |
| 28095 | M13 240189 | 11.2 | n/a | UK | 2013 | C | 5-1 | 10-8 | 11 |
| 28098 | M13 240280 | 11.2 | n/a | UK | 2013 | C | 5-1 | 10-8 | 11 |
| 28104 | M13 240405 | 11.2 | n/a | UK | 2013 | C | 5-1 | 10-8 | 11 |
| 26731 | M13 240559 | 11.2 | n/a | UK | 2013 | C | 5-1 | 10-8 | 11 |
| 30290 | M14 240187 | 11.2 | n/a | Canada | 2009 | C | 5-1 | 10-8 | 11 |
| 30295 | M14 240192 | 11.2 | n/a | Canada | 2012 | C | 7-1 | 1 | 11 |
| 30291 | M14 240188 | 11.2 | n/a | Canada | 2009 | C | 5-1 | 10-8 | 11 |
| 30294 | M14 240191 | 11.2 | n/a | Canada | 2011 | C | 7-1 | 1 | 11 |
|  |  |  |  |  |  |  |  |  |  |
| 1181 | W-138 | 11.2 | n/a | Greece | 1998 | B | 5 | 2 | 11 |
| 29982 | M98 251316 | 11.2 | n/a | UK | 1998 | B | 5 | 2 | 11 |
| 29984 | M99 240124 | 11.2 | n/a | UK | 1999 | B | 5 | 2 | 11 |
| 29988 | M00 240440 | 11.2 | n/a | UK | 2000 | B | 5 | 2 | 11 |
| 29993 | M01 240025 | 11.2 | n/a | UK | 2001 | B | 5 | 2 | 11 |
| 30047 | M02 240690 | 11.2 | n/a | UK | 2002 | B | 5 | 2 | 11 |
| 30296 | M14 240193 | 11.2 | n/a | Canada | 2001 | C | 5 | 2 | 11 |
| 30297 | M14 240194 | 11.2 | n/a | Canada | 2001 | C | 5 | 2 | 11 |
| 30298 | M14 240195 | 11.2 | n/a | Canada | 2001 | C | 5 | 2 | 11 |
| 30299 | M14 240196 | 11.2 | n/a | Canada | 2001 | C | 5 | 2 | 11 |
| 30300 | M14 240197 | 11.2 | n/a | Canada | 2001 | C | 5 | 2 | 11 |
| 30301 | M14 240198 | 11.2 | n/a | Canada | 2001 | C | 5 | 2 | 11 |
| 30284 | M14 240181 | 11.2 | n/a | Canada | 2002 | C | 5 | 2 | 11 |
| 1171 | BM48 | 11.2 | n/a | Greece | 1996 | C | 5 | 2 | 11 |
| 1169 | BM45 | 11.2 | n/a | Greece | 1996 | C | 5 | 2 | 11 |
| 1178 | W-72 | 11.2 | n/a | Greece | 1997 | C | 5 | 2 | 211 |
| 29904 | M98 251593 | 11.2 | n/a | Ireland | 1998 | C | 5 | 2 | 11 |
| 29918 | M99 241412 | 11.2 | n/a | Ireland | 1999 | C | 5 | 2 | 11 |
| 29924 | M99 243272 | 11.2 | n/a | Ireland | 1999 | C | 5 | 2 | 11 |
| 29609 | M05 241241 | 11.2 | n/a | Ireland | 2005 | C | 5 | 2 | 11 |
| 29605 | M05 240920 | 11.2 | n/a | Ireland | 2005 | C | 5 | 2 | 11 |
| 29287 | 2666 | 11.2 | n/a | South Africa | 2005 | C | 5 | 2 | 11 |
| 30232 | M96 252550 | 11.2 | n/a | UK | 1996 | C | 5 | 2 | 11 |
| 30191 | M96 255864 | 11.2 | n/a | UK | 1996 | C | 5 | 2 | 11 |
| 30190 | M96 255863 | 11.2 | n/a | UK | 1996 | C | 5 | 2 | 11 |
| 29738 | M97 252092 | 11.2 | n/a | UK | 1997 | C | 5 | 2 | 11 |
| 29740 | M97 252094 | 11.2 | n/a | UK | 1997 | C | 5 | 2 | 11 |
| 29739 | M97 252093 | 11.2 | n/a | UK | 1997 | C | 5 | 2 | 11 |
| 30171 | M98 252111 | 11.2 | n/a | UK | 1998 | C | 5 | 2 | 11 |
| 29912 | M98 253765 | 11.2 | n/a | UK | 1998 | C | 5 | 2 | 11 |
| 29902 | M98 251032 | 11.2 | n/a | UK | 1998 | C | 5 | 2 | 11 |
| 29908 | M98 252918 | 11.2 | n/a | UK | 1998 | C | 5 | 2 | 1055 |
| 29820 | M98 252927 | 11.2 | n/a | UK | 1998 | C | 5 | 2 | 11 |
| 29822 | M98 252947 | 11.2 | n/a | UK | 1998 | C | 5 | 2 | 11 |
| 29821 | M98 252928 | 11.2 | n/a | UK | 1998 | C | 5 | 2 | 11 |
| 29914 | M99 240413 | 11.2 | n/a | UK | 1999 | C | 5 | 2 | 3455 |
| 29922 | M99 242522 | 11.2 | n/a | UK | 1999 | C | 5 | 2 | 11 |
| 29920 | M99 242207 | 11.2 | n/a | UK | 1999 | C | 5 | 2 | 11 |
| 29833 | M00 240320 | 11.2 | n/a | UK | 2000 | C | 5 | 2 | 11 |
| 29836 | M00 240845 | 11.2 | n/a | UK | 2000 | C | 5 | 2 | 11 |
| 29838 | M00 241306 | 11.2 | n/a | UK | 2000 | C | 5 | 2 | 11 |
| 29666 | M00 241348 | 11.2 | n/a | UK | 2000 | C | 5 | 2 | 11 |
| 29844 | M00 243289 | 11.2 | n/a | UK | 2000 | C | 5 | 2 | 11 |
| 29859 | M01 242752 | 11.2 | n/a | UK | 2001 | C | 5 | 2 | 11 |
| 29674 | M01 241431 | 11.2 | n/a | UK | 2001 | C | 5 | 2 | 3455 |
| 29789 | M01 241286 | 11.2 | n/a | UK | 2001 | C | 5 | 2 | 11 |
| 29790 | M01 241302 | 11.2 | n/a | UK | 2001 | C | 5 | 2 | 11 |
| 29673 | M01 241306 | 11.2 | n/a | UK | 2001 | C | 5 | 2 | 11 |
| 29853 | M01 241276 | 11.2 | n/a | UK | 2001 | C | 5 | 2 | 11 |
| 29868 | M02 240945 | 11.2 | n/a | UK | 2002 | C | 5 | 2 | 11 |
| 29860 | M02 240005 | 11.2 | n/a | UK | 2002 | C | 5 | 2 | 11 |
| 29870 | M02 241124 | 11.2 | n/a | UK | 2002 | C | 5 | 2 | 11 |
| 30226 | M03 241190 | 11.2 | n/a | UK | 2003 | C | 5 | 2 | 11 |
| 29571 | M04 240932 | 11.2 | n/a | UK | 2004 | C | 5 | 2 | 11 |
| 29576 | M04 240065 | 11.2 | n/a | UK | 2004 | C | 5 | 2 | 11 |
| 29575 | M04 240029 | 11.2 | n/a | UK | 2004 | C | 5 | 2 | 11 |
| 29606 | M05 240972 | 11.2 | n/a | UK | 2005 | C | 5 | 2 | 11 |
| 29617 | M06 240375 | 11.2 | n/a | UK | 2006 | C | 5 | 2 | 11 |
| 29879 | M08 240142 | 11.2 | n/a | UK | 2008 | C | 5 | 2 | 11 |
| 29835 | M00 240680 | 11.2 | n/a | UK | 2000 | C | 21 | 16 | 7128 |
| 29590 | M04 241209 | 11.2 | n/a | UK | 2004 | C | 21 | 16 | 11 |
| 30060 | M09 240026 | 11.2 | n/a | UK | 2009 | B | 5-1 | 10-1 | 11 |
| 29671 | M00 243130 | 11.2 | n/a | UK | 2000 | B | 5-1 | 10-4 | 11 |
| 29598 | M05 240258 | 11.2 | n/a | Ireland | 2005 | C | 5-1 | 10-4 | 5170 |
| 644 | L93/4286 | 11.2 | n/a | UK | 1993 | C | 5-1 | 10-4 | 11 |
| 672 | 2847 | 11.2 | n/a | UK | 1997 | C | 5-1 | 10-4 | 50 |
| 662 | 2837 | 11.2 | n/a | UK | 1997 | C | 5-1 | 10-4 | 50 |
| 670 | 2840 | 11.2 | n/a | UK | 1997 | C | 5-1 | 10-4 | 50 |
| 671 | 2844 | 11.2 | n/a | UK | 1997 | C | 5-1 | 10-4 | 50 |
| 29899 | M98 250423 | 11.2 | n/a | UK | 1998 | C | 5-1 | 10-4 | 11 |
| 30202 | M99 240747 | 11.2 | n/a | UK | 1999 | C | 5-1 | 10-4 | 11 |
| 29655 | M99 241273 | 11.2 | n/a | UK | 1999 | C | 5-1 | 10-4 | 11 |
| 29832 | M00 240186 | 11.2 | n/a | UK | 2000 | C | 5-1 | 10-4 | 11 |
| 29834 | M00 240543 | 11.2 | n/a | UK | 2000 | C | 5-1 | 10-4 | 11 |
| 29843 | M00 243016 | 11.2 | n/a | UK | 2000 | C | 5-1 | 10-4 | 11 |
| 29672 | M01 240219 | 11.2 | n/a | UK | 2001 | C | 5-1 | 10-4 | 11 |
| 29979 | M03 241792 | 11.2 | n/a | UK | 2003 | C | 5-1 | 10-4 | 11 |
| 29592 | M04 241501 | 11.2 | n/a | UK | 2004 | C | 5-1 | 10-4 | 11 |
| 29641 | M08 240231 | 11.2 | n/a | UK | 2008 | C | 5-1 | 10-4 | 7979 |
| 29850 | M01 240514 | 11.2 | n/a | UK | 2001 | C | 7-2 | 4 | 5069 |
| 29875 | M03 240453 | 11.2 | n/a | UK | 2003 | C | 7-2 | 4 | 11 |
| 664 | 2838 | 11.2 | n/a | UK | 1997 | C | IS*1301* | 2 | 11 |
| 663 | 2839 | 11.2 | n/a | UK | 1997 | C | IS*1301* | 2 | 11 |
| 29656 | M99 241594 | 11.2 | n/a | UK | 1999 | C | NS | 2 | 11 |
| 29577 | M04 240078 | 11.2 | n/a | UK | 2004 | C | IS*1301* | 2 | 11 |
|  |  |  |  |  |  |  |  |  |  |
| 29831 | M98 252173 | 11.2 | n/a | UK | 1975 | B | 5 | 2 | 475 |
| 30257 | M02 242016 | 11.2 | n/a | UK | 1970 | B | 5 | 2 | 475 |
| 30261 | M02 242014 | 11.2 | n/a | UK | 1970 | B | 5-1 | 2-2 | 11 |
| 30260 | M02 242007 | 11.2 | n/a | UK | 1970 | C | 5 | 2 | 11 |
| 20261 | M11 240123 | 11.2 | n/a | UK | 2011 | B | 5-1 | 2-2 | 3537 |

* = *fumC* broken between contigs. NV = new variant. NS = nonsense mutation in VR1. IS*1301 =* Putatively interrupted by IS*1301*. Genomes highlighted yellow were used to represent lineage 11.1 in cgMLST comparison for figure 2.

**Supplementary table 2 – Geotemporal distribution of isolates by serogroup.**

| **Country** | **Number (years/range)** | | |
| --- | --- | --- | --- |
|  | **Serogroup B** | **Serogroup C** | **Serogroup W** |
| UK | 30 (1970 - 2013) | 190 (1970 - 2014) | 302 (1975 - 2014) |
| Ireland |  | 14 (1998 to 2011) | 3 (2013) |
| France |  | 4 (2007 - 2013) | 4 (2000 - 2014) |
| Spain |  | 6 (1985 - 2002) |  |
| Italy |  | 3 (1984 - 2008) |  |
| Greece | 2 (1996 - 1998) | 3 (1996 - 1997) |  |
| Malta |  | 4 (2006 - 2013) | 2 (1999) |
| Poland |  | 1 (2012) |  |
| Norway | 1 (1969) |  |  |
| Israel |  | 1 (1988) |  |
| Turkey |  |  | 3 (2005 - 2006) |
| South Africa |  | 2 (2005 - 2012) | 109 (2003 - 2013) |
| Ghana |  | 1 (1984) |  |
| Mali |  | 1 (1989) |  |
| Algeria |  |  | 2 (1999 - 2001) |
| Burkina Faso |  |  | 8 (2001 - 2004) |
| Cameroon |  |  | 7 (2000 - 2001) |
| Chad |  |  | 2 (1996 - 2001) |
| Niger |  |  | 11 (2001 - 2003) |
| Senegal |  |  | 3 (2000 - 2001) |
| USA | 1 (1964) | 1 (1983) |  |
| Canada |  | 14 (2001 - 2012) |  |
| Argentina |  |  | 5 (2008 - 2012) |
| Brazil |  | 1 (1976) | 7 (2008 - 2011) |
