# Supplementary material for: Genomic resolution of an aggressive, widespread, diverse and expanding meningococcal serogroup B, C and W lineage
Source: J Infect. 2015 Nov;71(5):544–52. doi: 10.1016/j.jinf.2015.07.007 (PMC4635312; doi:10.1016/j.jinf.2015.07.007)
Supplement: Supplementary file 2 [file mmc2.docx]

**Supplementary methods**

Selection of the isolates (genomes) used in the study was as follows:

**MenW:cc11 isolates**

For England, Wales and Northern Ireland, the MenW:cc11 study sample included a comprehensive recent collection of all 72 isolates from the MGL from July 2010 to June 2013 and a subset of 47 isolates from the following epidemiological year (2013-2014; to February). A further 29 recent serogroup W isolates from a UK carriage study^1^ were also included (29 donors across five sites; 2010-2011). In order to capture the majority of English, Welsh and Northern Irish MenW:cc11 isolates from March 2000 to July 2010, we sequenced all known MenW:cc11 and/or MenW:PorB 2a and/or MenW:PorB NT (non-typable):PorA P1.5,2 isolates from the MRU archive for the corresponding period (yielding 127 MenW:cc11 isolates). The majority of those excluded from this period had non-cc11 sequence types, uncharacteristic PorB types and/or PorA subtypes characteristic of MenW:cc22 (e.g. PorA P1.18-1,3). No MenW:cc11 isolates had previously been identified by MLST on representative panels from 1985 or 1995^2^ so a small selection of 14 potential MenW candidates were sequenced based on the presence of PorB (2a) and/or PorA P1.5,2 (1987 to 1998; all were MenW:cc11). A further six previously identified MenW:cc11 isolates from a panel of 125 MRU isolates from 1975^2^ were also sequenced as well as a further 12 miscellaneous isolates from the UK, Ireland and Malta (1999-2014).

From other countries, a comprehensive panel of 109 MenW:cc11 isolates from South Africa (2003 to 2013) were included having been identified from a random selection of ten to fifteen MenW isolates per year that had been sequenced to yield 116/125 MenW:cc11 isolates. Smaller MenW:cc11 samples sequenced specifically for this study included four recent French isolates (2014), two isolates from confirmed French Hajj pilgrims (2000), five Argentinean isolates (2008-2012), seven Brazilian isolates (2008 2011), three Turkish isolates (2005-6), and a panel of 33 North African isolates (31 of which occurred between 2000 and 2004).

**MenC:cc11 isolates**

From England, Wales and Northern Ireland, the MenC:cc11 study sample included a comprehensive recent collection of all 35 Men:cc11 isolates from the MGL (July 2010 to June 2013) and a subset of six isolates from the following epidemiological year (2013-2014, up to March 2014). In order to capture the majority of English, Welsh and Northern Irish MenC:cc11 isolates from 2004 to July 2010 (inclusive), we sequenced all 94 known or suspected MenC:cc11 isolates (based on the presence of PorB 2a) of which 51 sequences (all cc11) were available at the time of analysis. Approximately 15 isolates per year from 2008 to 2003 (inclusive) were also selected to represent the PorA distribution of MenC PorB 2a isolates for the respective years. At the time of analysis 48 out 89 of these sequences (all cc11) were available. All seven MenW:cc11 isolates previously identified by MLST on a representative panel from 1985 were sequenced. As none had previously been identified in 1975, four known MenW:cc11 isolates from 1970 were sequenced. A further panel of 39 miscellaneous MenC:cc11 genomes from the UK (1996 to 2002) were also included.

From other countries, isolates specifically sequenced for this project included six Canadian serogroup C ET-15 isolates representing three distinct PorA subtypes (2001-2012) and two Canadian serogroup C ET-37 (non-ET-15) isolates (2008-2009), six Canadian isolates from an outbreak among MSM (2001), five Spanish ET-15 isolates (1999-2002) and miscellaneous isolates from Ireland (n=14, 1998-2011), Malta (n=4, 2006-2013), France (n=4, 2007-2013), Spain (n=1, 1985), Italy (n=3, 1984-1008), Greece (n=3, 1996-1997), Poland (n=1, 2012), Israel (n=1, 1988), Ghana (n=1, 1984), Mali (n=1, 1989), South Africa (n=2, 2005-2012), USA (n=1, 1983) and Brazil (n=1, 1976).

**MenB:cc11 isolates**

From England, Wales and Northern Ireland, the MenB:cc11 study sample included a comprehensive recent collection of all ten serogroup B cc11 isolates from the MGL (July 2010 to June 2013), previously identified cc11 isolates from 1975 (n=1) and 1970 (n=2) and 17 miscellaneous serogroup B PorB 2a isolates from 1998 to 2009. A further four miscellaneous isolates were from Greece (n=2, 1996 to 1998), Norway (n=1, 1969) and the USA (n=1, 1964).

1. Read RC, Baxter D, Chadwick DR, et al. Effect of a quadrivalent meningococcal ACWY glycoconjugate or a serogroup B meningococcal vaccine on meningococcal carriage: an observer-blind, phase 3 randomised clinical trial. *Lancet* 2014; **384**(9960): 2123-31.

2. Russell JE, Urwin R, Gray SJ, Fox AJ, Feavers IM, Maiden MC. Molecular epidemiology of meningococcal disease in England and Wales 1975-1995, before the introduction of serogroup C conjugate vaccines. *Microbiology* 2008; **154**(Pt 4): 1170-7.
